# SPP1, LYZ, and MCM5: potential diagnostic biomarkers for rheumatoid arthritis and cervical cancer comorbidity

**DOI:** 10.3389/fmed.2025.1693787

**Published:** 2025-11-26

**Authors:** Xiaoyang Liu, Wenhao Wang, Yuhui Zhao, Xueying Gu, Ruihe Wu, Kaili Qin, Xiaofeng Li, Chong Gao, Caihong Wang

**Affiliations:** 1 Department of Rheumatology, The Second Hospital of Shanxi Medical University, Taiyuan, Shanxi, China; 2 Shanxi Key Laboratory of Rheumatism Immune Microecology, Taiyuan, Shanxi, China; 3 Shanxi Precision Medical Engineering Research Center for Rheumatology, Taiyuan, Shanxi, China; 4 Department of Obstetrics and Gynecology, The Second Hospital of Shanxi Medical University, Taiyuan, Shanxi, China; 5 Department of Pathology, Brigham and Women's Hospital/Children's Hospital Boston, Joint Program in Transfusion Medicine, Harvard Medical School, Boston, MA, United States

**Keywords:** rheumatoid arthritis, cervical cancer, human papillomavirus, autoimmunity, tumor immunity, diagnostic biomarkers

## Abstract

**Background:**

The comorbidity of rheumatoid arthritis (RA), a chronic autoimmune disease, with cervical cancer has garnered a lot of attention. Cervical cancer is much more common in RA patients than in the general population, which may be caused by immunosuppressive therapy, chronic inflammation, and poor clearance of the Human Papillomavirus (HPV). The purpose of this study is to explore the molecular mechanism of comorbidity between RA and cervical cancer and identify potential biomarkers through transcriptomics and single cell transcriptomics analysis.

**Methods:**

In this study, transcriptome expression profile data of RA and cervical cancer were downloaded from Gene Expression Omnibus (GEO) and The Cancer Genome Atlas (TCGA) databases, and differential gene analysis, Gene Ontology (GO) and Kyoto Encyclopedia of Genes and Genomes (KEGG) functional enrichment analysis were performed. Using multivariate Cox proportional hazard modeling and Lasso regression, independent differential genes linked to the prognosis of cervical cancer were screened. Molecular docking technology was used to predict the interaction between candidate gene encoded proteins and HPV 16 E6/E7. Intercellular communication and the expression patterns of potential genes in various cell groups were examined using single cell transcriptome data. Finally, the expression of candidate genes in cervical tissues of patients with RA combined with cervical cancer was verified by immunohistochemistry.

**Results:**

The study found that those with RA had 493 up-regulated genes and 216 down-regulated genes, while individuals with cervical cancer had 2,600 up-regulated genes and 2,172 down-regulated genes. Cox regression analysis identified 35 genes independently associated with the prognosis of cervical cancer, of which SPP1, LYZ, and MCM5 were significantly regulated in both RA and cervical cancer. The HPV 16 E6/E7 specific binding sites of the proteins produced by these three genes were shown using molecular docking simulation. Especially, single cell transcriptomic analysis revealed that SPP1 was highly expressed in NK/T cells, Myeloid cells, and epithelial cells, and served as an important ligand receptor pair for communication between these cells. Immunohistochemistry results further verified the high expression of SPP1, LYZ, and MCM5 in patients with RA combined with cervical cancer.

**Conclusion:**

This study successfully identified SPP1, LYZ, and MCM5 as key hub genes for the comorbidity of RA and cervical cancer. By regulating processes like inflammation, immune evasion, and cell proliferation, these genes not only have a high diagnostic potential but may also contribute to the occurrence and development of cervical cancer.

## 1 Introduction

Rheumatoid arthritis (RA) is an autoimmune disease characterized by chronic, symmetrical polyarticular inflammation, which can lead to joint destruction, loss of function, and systemic damage to multiple systems (1, 2). Cervical cancer is the fourth most common cancer in women worldwide, with more than 600,000 new cases and about 340,000 deaths each year, and more than 85% of them are concentrated in low- and middle-income countries with scarce medical resources. An incidence rate of 13.8/100,000, a mortality rate of 4.5/100,000, and 56,000 deaths were recorded in China in 2022, with 151,000 new cases of cervical cancer (3, 4). Cervical cancer’s age standardized disability adjusted life year rate is 110 DALYs/100,000 (5). The burden is heaviest among working age women aged 30–59. Direct medical expenses and productivity losses due to cervical cancer in low- and middle-income countries account for 0.5–1.2% of total annual health expenditures (6). The incidence of cervical cancer in RA patients is 1.5–2 times greater than in the general population, according to the latest studies (7). This could be because of immunosuppressive therapy, chronic inflammation, and poor clearance of the Human Papillomavirus (HPV) (8, 9). Related mechanism studies have found that RA related proinflammatory factors may activate HPV oncogenes and promote malignant transformation of cervical epithelial cells by activating signaling pathways. Immunosuppressive therapy can weaken the anti-HPV response of local cervical immune cells and accelerate disease progression (10–12). All of these will significantly increase the risk of an ongoing HPV infection in RA patients and delay HPV clearance (13). Cervical cancer has also been confirmed to be a cancer driven by persistent infection with high-risk HPV (HR-HPV), but not all women infected with HPV will develop cervical cancer (8). Obviously, the occurrence of cervical carcinogenesis must be synergistic with other factors besides HR-HPV infection (14). Exploring the synergistic carcinogenic factors and mechanisms of action of HPV will help to improve the occurrence mechanism of cervical cancer. In addition, in comorbidity research, transcriptomics can reveal the interaction between diseases and become a powerful tool for cross research on immune diseases (15–17). This evidence suggests that the study of comorbidity between RA and cervical cancer is an intersectional field connecting autoimmunity and tumor immunity. Its results will reshape the prevention and control path of high-risk populations and provide a paradigm for the exploration of the mechanism of chronic inflammation related cancer.

This study identified shared genes that are differently expressed in cervical cancer and RA using transcriptomics etc. We further assessed the clinical significance of these genes in cervical cancer patients and investigated the potential molecular underpinnings of the formation of chronic inflammation related cervical cancer by examining the interaction between these genes in immune cells and epithelial cells. Furthermore, we explored the molecular mechanism of comorbidity between RA and cervical cancer by immunohistochemistry analysis of patients with cervical cancer combined with RA, and successfully identified three key hub genes: SPP1, LYZ, and MCM5.

## 2 Materials and methods

### 2.1 Data collection

**Figure 1:**
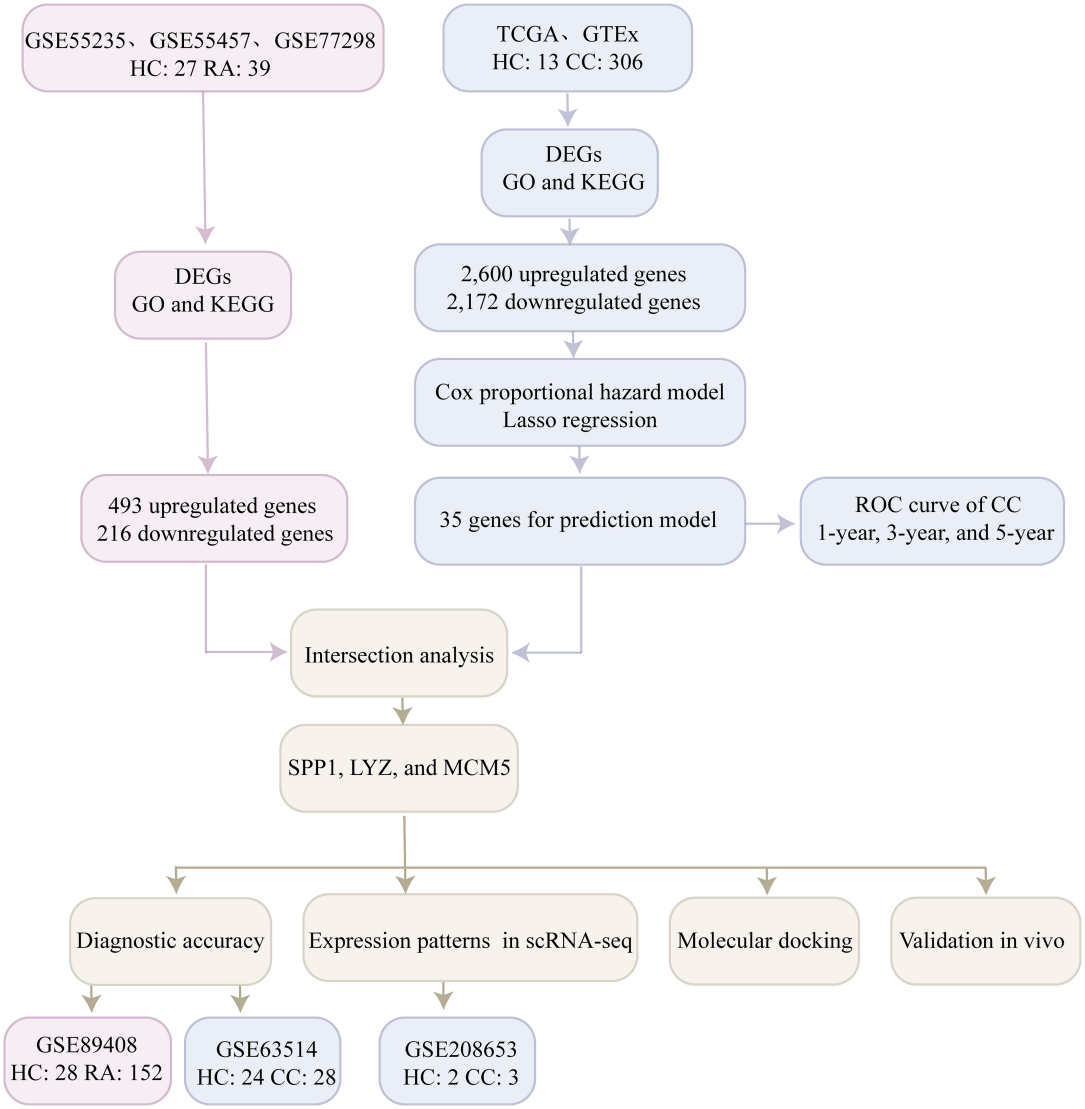
Flowchart illustrating the main methods of the current study. Healthy controls, HC; Rheumatoid arthritis, RA; Cervical cancer, CC; Differentially expressed genes, DEGs; Receiver operating characteristic curve, ROC; Gene ontology, GO; Kyoto Encyclopedia of Genes and Genomes, KEGG; Single cell RNA Sequencing, scRNA-seq.

“Rheumatoid arthritis” and “cervical cancer” were used as search terms in the Gene Expression Omnibus (GEO) database to find the transcriptome expression profile data. The four RA datasets downloaded from GEO were GSE55235 (healthy controls, 10 cases; RA, 10 cases), GSE55457 (healthy controls, 10 cases; RA, 13 cases) (18), GSE77298 (healthy controls, 7 cases; RA, 16 cases) (19) and GSE89408 (healthy controls, 28 cases; RA, 152 cases) (20), and the cervical cancer dataset was GSE63514 (healthy controls, 24 cases; cervical cancer, 28 cases) (21). In addition, we downloaded the expression profile dataset of cervical cancer (healthy controls, 3 cases; cervical cancer, 306 cases) from the TCGA database,^1^ and the expression profile dataset of normal cervical tissue (healthy controls, 10 cases) from Genotype Tissue Expression (GTEx) (22). Finally, the single cell transcriptome dataset of cervical cancer GSE208653 (healthy control, 2 cases; cervical cancer, 3 cases) (23) was downloaded from GEO for analysis. The workflow of this study is shown in Figure 1.

### 2.2 Differential gene analysis

The three RA datasets GSE55235, GSE55457 and GSE77298 were merged based on the remove_batcheffect function of the IOBR2 R package (24), and the limma R package (25) was used for analysis of differential genes. Principal component analysis (PCA) revealed a more uniform sample distribution following the removal of batch effects with IOBR2, indicating their effective removal (Supplementary Figures S1a,b). The TCGA and GTEx datasets were then combined and made correct, differential gene analysis was performed using the R language’s edgeR (26) package, and the differential gene heat map was created using the pheatmap package. The standard screening criteria for differentially expressed genes (DEGs) are logFC > 1 or < −1 and adj. *p* value < 0.05. We classified logFC > 1 or < −1 as up-regulated or down-regulated.

### 2.3 GO and KEGG functional enrichment analysis of differentially expressed genes

Gene function [biological process (BP), cell component (CC), and molecular function (MF)] and KEGG functional enrichment analysis were conducted on differentially expressed genes using the clusterProfiler (27) software package (v4.0). For multiple test correction, the Benjamini-Hochberg technique was employed, and a difference was deemed statistically significant when *p* < 0.05.

### 2.4 Hub gene screening and validation

We first used the univariate Cox proportional hazard model to screen differentially expressed genes significantly associated with cervical cancer overall survival (OS) and then screened differentially expressed genes using the Lasso regression algorithm and then used the multivariate Cox proportional hazard model to screen independent differentially expressed genes associated with cervical cancer OS. The study made use of the Survminer R package’s surv_cutpoint function to calculate the cutoff value of cervical cancer risk factors and divided the results into a low risk group (risk score less than the cutoff value) and a high risk group (risk score greater than or equal to the cutoff value). Finally, using the time dependent receiver operating characteristic curve (ROC), the model’s predictive ability was confirmed in the training set.

### 2.5 Diagnostic evaluation of candidate hub genes in RA and cervical cancer

We intersected the differentially expressed genes of RA with the genes associated with the prognosis of cervical cancer in order to confirm the diagnostic accuracy of hub genes in RA and cervical cancer. In order to assess the diagnostic utility of candidate hub genes in RA and cervical cancer, the study compiled the area under the curve (AUC) in the test and validation sets for the common differentially expressed genes of RA and cervical cancer using the pROC (28) R language package.

### 2.6 Molecular docking simulation of candidate hub genes and HPV E6/E7

We obtained the three dimensional protein structures of HPV16 E6, HPV16 E7, SPP1, LYZ and MCM5 from the Protein Data Bank (PDB). Then, the interaction network between the candidate gene encoded proteins and HPV16 E6/E7 was systematically analyzed by molecular docking technology. The study predicted the three dimensional structure of related proteins based on the AlphaFold 3 deep learning platform. PyMOL was used to remove water molecules and original ligands from the target protein, and AutoDockTools was used to perform hydrogenation, charge calculation, and nonpolar hydrogen bonding. After the grid parameters and genetic algorithm were determined, AutoDock Vina was used for molecular docking, and the results were finally visualized using Discovery Studio and PyMOL.

### 2.7 Single cell transcriptomics data processing

We used Seurat (v5.1) (29) for further analysis based on the cervical cancer single cell transcriptome dataset (GSE208653). The study’s quality control parameters were set as more than 500 genes in the cell, more than 4,000 RNA counts per cell, and less than 20% of mitochondrial reads. Following the filtering process, the expression matrix was transformed [ln (CPM + 1)] and normalized using the NormalizeData function in the Seurat package. With highly variable genes as input, principal component analysis was then performed using “RunPCA.” A graph-based clustering algorithm was used for the clustering process, and the Seurat function “Run tSNE” was used for visualization. To deal with batch effects between data sets, we used the Harmony method for data integration. PCA and t-distributed stochastic neighbor embedding (t-SNE) revealed a more uniform sample distribution following the removal of batch effects with Harmony, indicating their effective removal (Supplementary Figures S2a,b). We divided the cell populations into 9 groups according to marker genes: NK/T cells, Neutrophils, Epithelial cells, Myeloid cells, Plasma Cells, Mast cells, B cells, Fibroblasts, and Endothelial cells. We then used DotPlot and FeaturePlot functions to analyze the expression patterns of the three candidate genes in different cell populations. To study the communication between ligands and receptors between cell clusters, Cellchat was used to analyze intercellular communication. Furthermore, we explored the cell–cell network between epithelial and myeloid cells and compared the differences in signaling pathways between cervical cancer and healthy controls.

### 2.8 Patient collection

The study retrospectively collected a total of 32 cases of cervical tissue pathological sections from patients at the Second Hospital of Shanxi Medical University between January 2021 and December 2024. Of these, 11 were healthy controls, 12 had cervical cancer, and 9 had RA combined with cervical cancer. The selection criteria for cervical cancer were as follows: cervical cancer was confirmed by pathology, and there was no history of chemoradiotherapy before surgery. In addition, diabetes, hypertension, and a history of malignant tumors were not included, nor were any other chronic systemic disorders. Patients with RA were diagnosed according to the 2010 ACR/EULAR RA diagnosis and classification criteria. Cervical tissue samples of healthy controls were obtained from persons who had a hysterectomy due to adenomyosis or uterine fibroids, and histology confirmed that the cervix had normal morphology and clear surgical margins. Detailed clinical characteristics of the three groups, including age, RA disease duration, treatment history (especially immunosuppressive therapy), and HPV infection status, are shown in Supplementary Table S1.

In accordance with the 1964 Declaration of Helsinki and its subsequent revisions or similar ethical standards, this study was approved by the Ethics Committee of the Second Hospital of Shanxi Medical University (approval number: 2023YX179). All subjects signed written informed consent.

### 2.9 Immunohistochemistry

We used an immunohistochemical staining kit (elabscience, China, E-IR-R215) to stain paraffin sections. First, the tissue sections were dewaxed, hydrated, antigen repaired, endogenous enzymes inactivated, and serum blocked. Then, SPP1 (Zenbio, China, 680476, 1:100), LYZ (Zenbio, China, 381103, 1:100), and MCM5 (Zenbio, China, R22573, 1:100) were dripped on the sections and incubated at 37 °C for 2 h. After washing with PBS three times, the secondary antibody (elabscience, China, E-IR-R215B) was dripped again, 30 min of incubation at 37 °C, followed by three PBS washes. Finally, DAB color developing solution was dripped on the sections, and brown-yellow was a positive result. ImageJ was used to quantify pathological images.

### 2.10 Statistical analysis

All data in this study were based on at least 3 biological replicates, and all statistical studies used R software (version 4.4.1). Measurement data are presented as the mean ± standard deviation. Intergroup comparisons were performed using one-way analysis of variance (ANOVA). If the ANOVA indicated a statistically significant difference, the Least Significant Difference (LSD) *post hoc* test was used for pairwise comparisons. To control for type I error inflation due to multiple comparisons, all *p* values were adjusted using the Bonferroni correction. If there was heterogeneity of variance, the Mann–Whitney U test was used. All *p* values were determined using two-sided testing, and *p* < 0.05 was taken as statistically significant.

## 3 Results

### 3.1 Identification of differentially expressed genes in patients with rheumatoid arthritis

**Figure 2:**
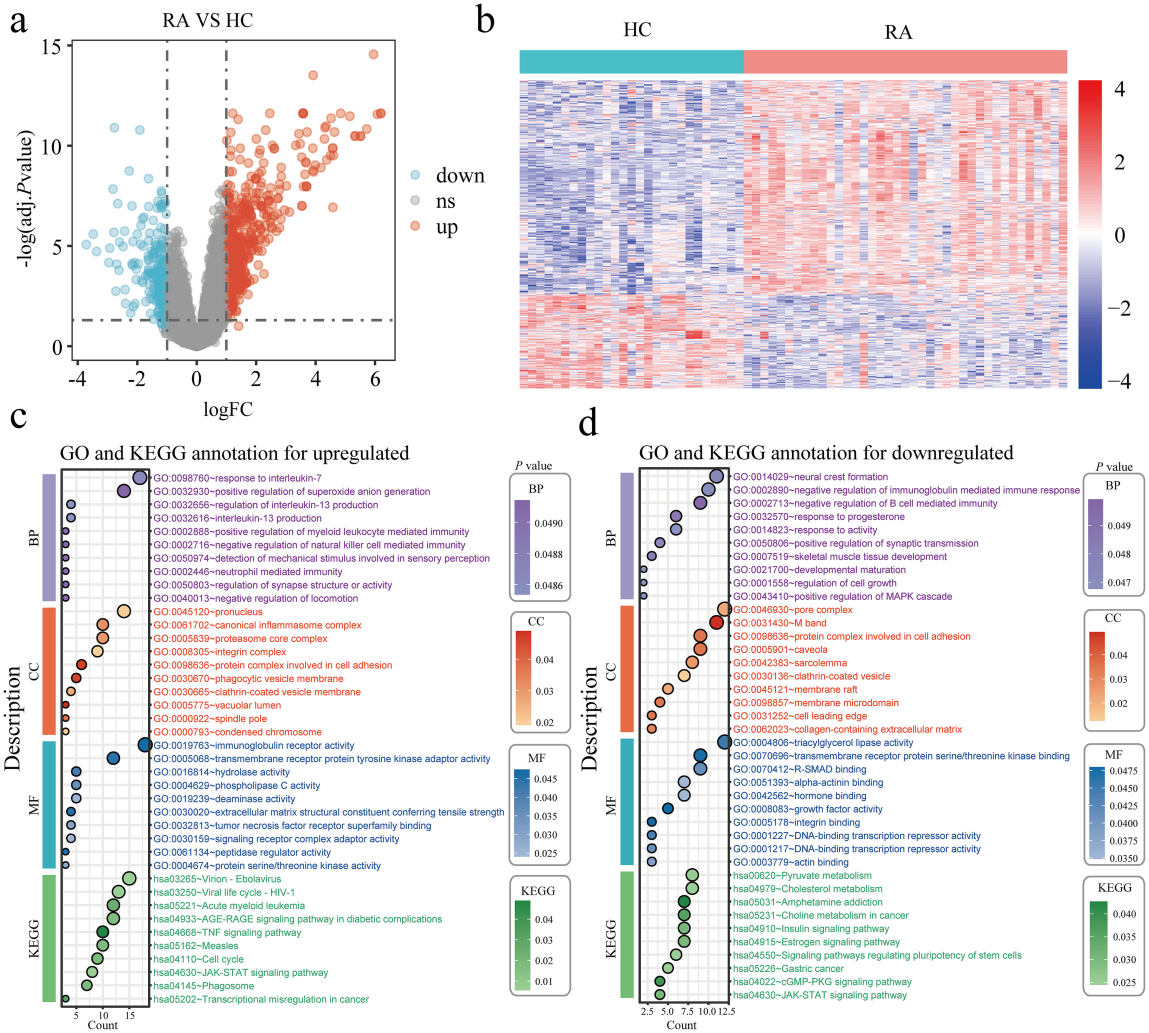
Identification of DEGs in rheumatoid arthritis patients. **(a)** Volcano plots of all DEGs in the integrated GSE55235, GSE55457, and GSE77298, upregulated DEGs are indicated by red, while downregulated DEGs are indicated by blue. **(b)** In the integrated GSE55235, GSE55457, and GSE77298 heatmap, the top DEGs are displayed. **(c,d)** The findings of the KEGG and GO enrichment analyses in upregulated **(c)** and downregulated **(d)** DEGs. Healthy controls, HC; Rheumatoid arthritis, RA; Differentially expressed genes, DEGs; Gene ontology, GO; Kyoto Encyclopedia of Genes and Genomes, KEGG.

We conducted differentially expressed gene analysis on the combined RA dataset (GSE55235, GSE55457, and GSE77298), which included 27 controls and 39 patients with rheumatoid arthritis. When the conditions were set as logFC > 1 or logFC < −1, and adj. *p* value < 0.05, we found that compared with the healthy control group, the rheumatoid arthritis group had 493 upregulated genes and 216 downregulated genes. We displayed the differentially expressed genes using heat maps and volcano plots (Figures 2a,b). GO and KEGG enrichment analyses were then conducted on the differential genes that were up-regulated and down-regulated (Figures 2c,d). The GO and KEGG enrichment of the up-regulated differentially genes showed that these up-regulated differentially genes were mainly concentrated in response to interleukin 7, neutrophil mediated immunity, negative regulation of natural killer cell mediated immunity, canonical inflammasome complex, immunoglobulin receptor activity, pathway for JAK–STAT signaling, cell cycle, and tumor necrosis factor signaling. GO and KEGG enrichment of the differentially down-regulated genes revealed their associations with the following: negative regulation of immunoglobulin mediated immune response, negative regulation of B cell mediated immunity, positive regulation of the MAPK cascade, regulation of cell growth, DNA binding transcription repressor activity, signaling pathways regulating stem cell pluripotency, JAK–STAT signaling pathway, and cGMP-PKG signaling pathway; chemical pathways involving estrogen, choline in cancer, amphetamine dependency, cholesterol, and pyruvate metabolism.

### 3.2 Identification of differentially expressed genes in cervical cancer patients

**Figure 3:**
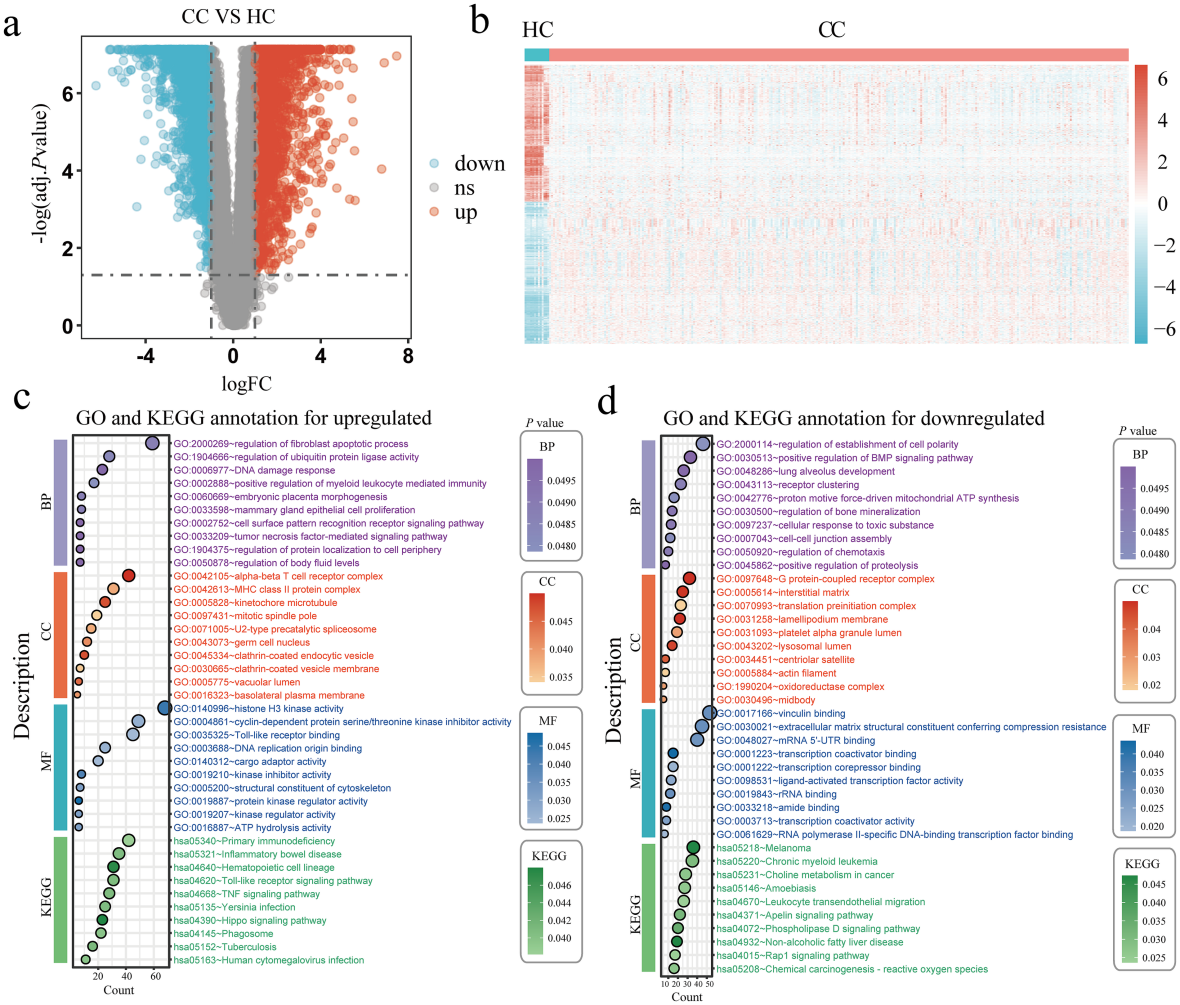
Identification of DEGs in cervical cancer. **(a)** Volcano plots of every DEG in the GTEx and TCGA; red denotes upregulated DEGs and blue denotes downregulated DEGs. **(b)** A heatmap of the top DEGs in the TCGA and GTEx. **(c,d)** The enrichment analysis results of GO and KEGG pathway in upregulated **(c)** and downregulated **(d)** DEGs. Healthy controls, HC; Cervical cancer, CC; Differentially expressed genes, DEGs; Gene ontology, GO; Kyoto Encyclopedia of Genes and Genomes, KEGG.

This study performed a joint analysis of the TCGA and GTEx databases, involving 13 healthy controls and 306 cervical cancer patients, to identify differentially expressed genes associated with cervical cancer. We set the differential gene screening conditions as logFC > 1 or logFC < −1, and adj. *p* value < 0.05. Compared with the healthy control group, we finally screened out 2,600 upregulated genes and 2,172 downregulated genes (Figures 3a,b). GO and KEGG enrichment of the differentially expressed genes that were up-regulated and down-regulated revealed that the majority of the up-regulated genes were involved in the regulation of the fibroblast apoptotic rate, regulation of ubiquitin protein ligase activity, DNA damage response, positive regulation of myeloid leukocyte mediated immunity, cell surface pattern recognition receptor signaling pathway, tumor necrosis factor mediated signaling pathway, alpha beta T cell receptor complex, MHC class II protein complex, histone H3 kinase activity, cyclin dependent protein serine/threonine kinase inhibitor activity, DNA replication origin binding, TNF signaling pathway, Toll like receptor signaling pathway and Hippo signaling pathway (Figure 3c). The majority of the differentially expressed genes that were down-regulated were involved in the positive regulation of the BMP signaling pathway, receptor clustering, proton motive force driven mitochondrial ATP synthesis, G protein coupled receptor complex, vinculin binding, and extracellular matrix structural elements that provide compression resistance, mRNA 5’ UTR binding, transcription coactivator and corepressor binding, choline metabolism in cancer, leukocyte transendothelial migration, apelin signaling pathway, Rap1 signaling pathway, phospholipase D signaling pathway (Figure 3d).

### 3.3 Using Lasso regression and Cox regression to identify hub genes associated with cervical cancer prognosis

**Figure 4:**
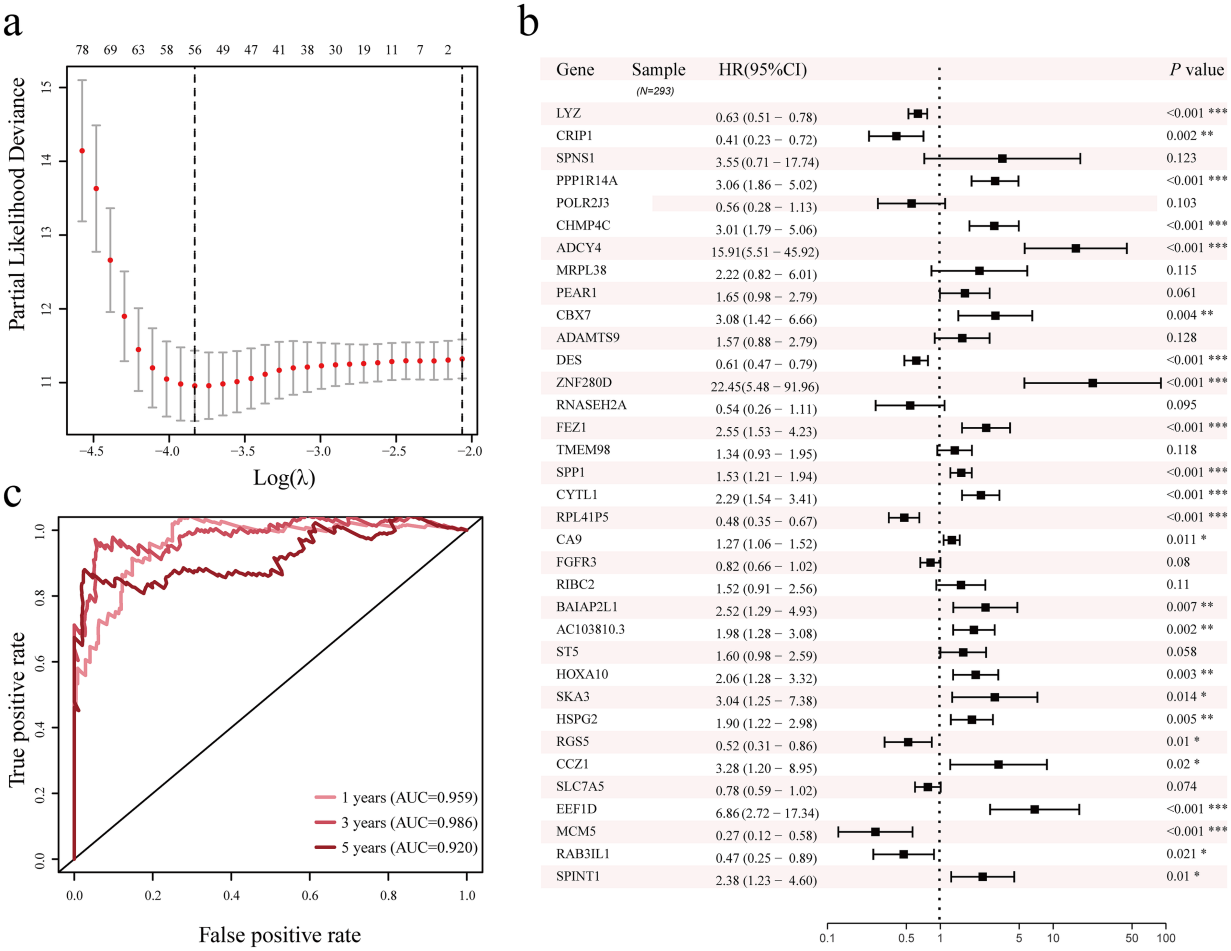
Lasso regression, univariate and multivariate Cox proportional models were used to identify genes associated with the prognosis of cervical cancer. **(a)** LASSO regression analysis of genes related to prognosis of cervical cancer. **(b)** Screening of genes related to prognosis of cervical cancer using multivariate Cox proportional model. **(c)** Analysis of the 1-year, 3-year, and 5-year prediction ability of the 35 genes cervical cancer prediction model. Area Under Curve, AUC.

The study first screened for differentially expressed genes linked to the prognosis of cervical cancer using a univariate Cox proportional model. The findings indicated that 199 genes with differential expression were linked to the prognosis of cervical cancer (*p* < 0.05, Supplementary Table S2). After that, we further screened prognostic genes using Lasso regression; 56 prognostic genes were included when *λ* = 0.022 (Figure 4a; Supplementary Figure S3). Afterwards, we further included the 56 genes in the multivariate Cox proportional model to further screen genes independently associated with the cervical cancer prognosis. The outcomes demonstrated 35 genes were screened and selected as prognostic predictors, that LYZ, CRIP1, PPP1R14A, CHMP4C, ADCY4, CBX7, DES, ZNF280D, FEZ1, SPP1, CYTL1, RPL41P5, CA9, BAIAP2L1, AC103810.3, HOXA10, SKA3, HSPG2, RGS5, CCZ1, EEF1D, MCM5, RAB3IL1, and SPINT1 were independent variables for cervical cancer development and occurrence (*p* < 0.05, Figure 4b). For the 1-, 3-, and 5-year prognoses of cervical cancer, we further assessed the predictive power of the prediction model built with 35 genes using the receiver operating characteristic curve (ROC). The areas under the ROC curve of the 35 gene prediction model for 1-year, 3-year, and 5-year cervical cancer overall survival prognosis were 0.959, 0.986, and 0.920 (Figure 4c), respectively, which also shows that the 35 genes used to develop the prediction model has a good predictive value in cervical cancer.

### 3.4 Diagnostic potential of candidate biomarkers in rheumatoid arthritis and cervical cancer

**Figure 5:**
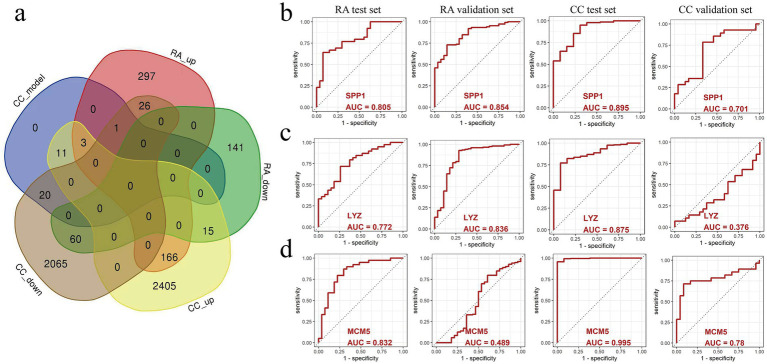
The predictive value of SPP1, LYZ, and MCM5 in relation to cervical cancer and rheumatoid arthritis (RA) was examined using a ROC curve. **(a)** The intersection of genes that are expressed differently in cervical cancer and RA. **(b)** ROC curves for evaluating the predictive value of SPP1 in the RA test dataset, RA validation dataset, cervical cancer test dataset, and cervical cancer validation dataset. **(c)** ROC curves for evaluating the predictive value of LYZ in the RA test dataset, RA validation dataset, cervical cancer test dataset, and cervical cancer validation dataset. **(d)** ROC curves for evaluating the predictive value of MCM5 in the RA test dataset, RA validation dataset, cervical cancer test dataset, and cervical cancer validation dataset. Healthy controls, HC; Rheumatoid arthritis, RA; Cervical cancer, CC; Area Under Curve, AUC.

By performing intersection analysis on 35 cervical cancer prognosis related genes and RA differential genes, a total of 3 co-upregulated differential genes were identified (Figure 5a), namely SPP1, LYZ, and MCM5, which were upregulated in patients with rheumatoid arthritis and cervical cancer. Next, we evaluated the three genes’ potential for diagnosis, SPP1, LYZ, and MCM5, in the RA test set data (GSE55235, GSE55457, and GSE77298) and validation set (GSE89408) as well as the cervical cancer test data sets (TCGA and GTEx) and validation set (GSE63514). The ROC curves showed that SPP1 had good diagnostic potential in the RA test set (AUC = 0.805), validation set (AUC = 0.854), and cervical cancer test set (AUC = 0.895) and validation set (AUC = 0.701) (Figure 5b); LYZ had good diagnostic potential in the RA test set (AUC = 0.772) and validation set (AUC = 0.836) and cervical cancer test set (AUC = 0.875) (Figure 5c); MCM5 also had good diagnostic potential in the RA test set (AUC = 0.832) and cervical cancer test set (AUC = 0.995) and validation set (AUC = 0.780) (Figure 5d). These results further suggest that SPP1, LYZ, and MCM5 may serve as potential diagnostic markers for patients with RA and cervical cancer. To explore the potential functional partnerships among SPP1, LYZ, and MCM5, we constructed a protein–protein interaction (PPI) network using the STRING database.^2^ Network analysis revealed that B2M (Beta 2 microglobulin) is a predicted common interactor for all three proteins, suggesting that B2M may serve as a functional hub mediating their collective role in the comorbid mechanism between RA and cervical cancer (Supplementary Figure S4).

### 3.5 Prediction of binding sites between candidate biomarkers and HPV 16 E6/E7 virus using molecular docking

**Figure 6:**
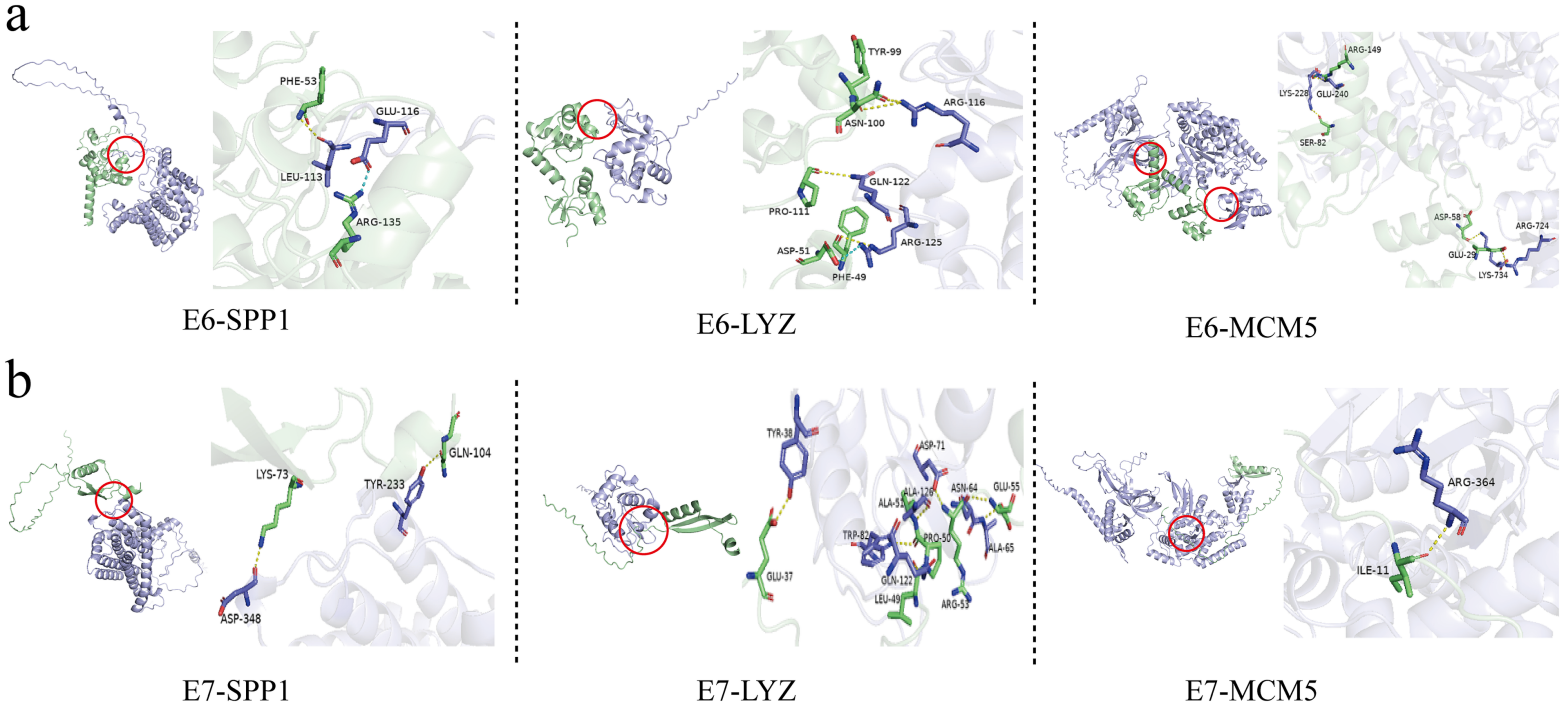
Molecular docking simulation between the proteins encoded by three candidate genes, SPP1, LYZ, and MCM5, and HPV16 E6/E7 viral proteins. **(a)** Molecular docking simulation between the proteins encoded by three candidate genes, SPP1, LYZ, and MCM5, and HPV16 E6 viral protein. **(b)** Molecular docking simulation between the proteins encoded by three candidate genes, SPP1, LYZ, and MCM5, and HPV16 E7 viral protein.

The study used computer simulation molecular docking to further study the interaction between three candidate biomarkers SPP1, LYZ, and MCM5 and HPV 16 E6/E7 proteins. Affinity refers to the ability of a ligand to bind to a receptor. The larger the absolute value of the affinity, the stronger the binding ability (note that the affinity value is negative). The respective binding energies of SPP1, LYZ, and MCM5 to E6 are −47.35, −27.65, and −12.89. SPP1 and E6 are bound by hydrogen bonds between Leu113 and Phe53, and Glu116 and Arg135 are bound by salt bridges (Figure 6a); LYZ and E6 are bound by hydrogen bonds between Arg116 and Asn100 and Gln122 and Pro111, and Arg125 and Asp51 are bound by salt bridges (Figure 6a); MCM5 and E6 are bound by hydrogen bonds between Glu240 and Arg149, Lys228 and Ser82, Lys734 and Asp58, and Arg724 and Glu29 (Figure 6a). In addition, we also compared the binding patterns of the proteins translated by these three candidate biomarkers with HPV16 E7. The respective binding energies of SPP1, LYZ, and MCM5 to E7 are −33.2, −120.84, and −54.69. The predicted patterns showed that SPP1 bound to Lys73 of E7 through Asp348 and Tyr233 to Gln104 through hydrogen bonds (Figure 6b); LYZ bound to E7 through Tyr38 to Glu37, Ala65 to Glu55, Asp71 to Arg53, etc. through hydrogen bonds (Figure 6b); MCM5 bound to Ile11 of E7 through Arg364 binds by hydrogen bonds (Figure 6b).

### 3.6 Expression patterns of SPP1, LYZ and MCM5 in scRNA-seq

**Figure 7:**
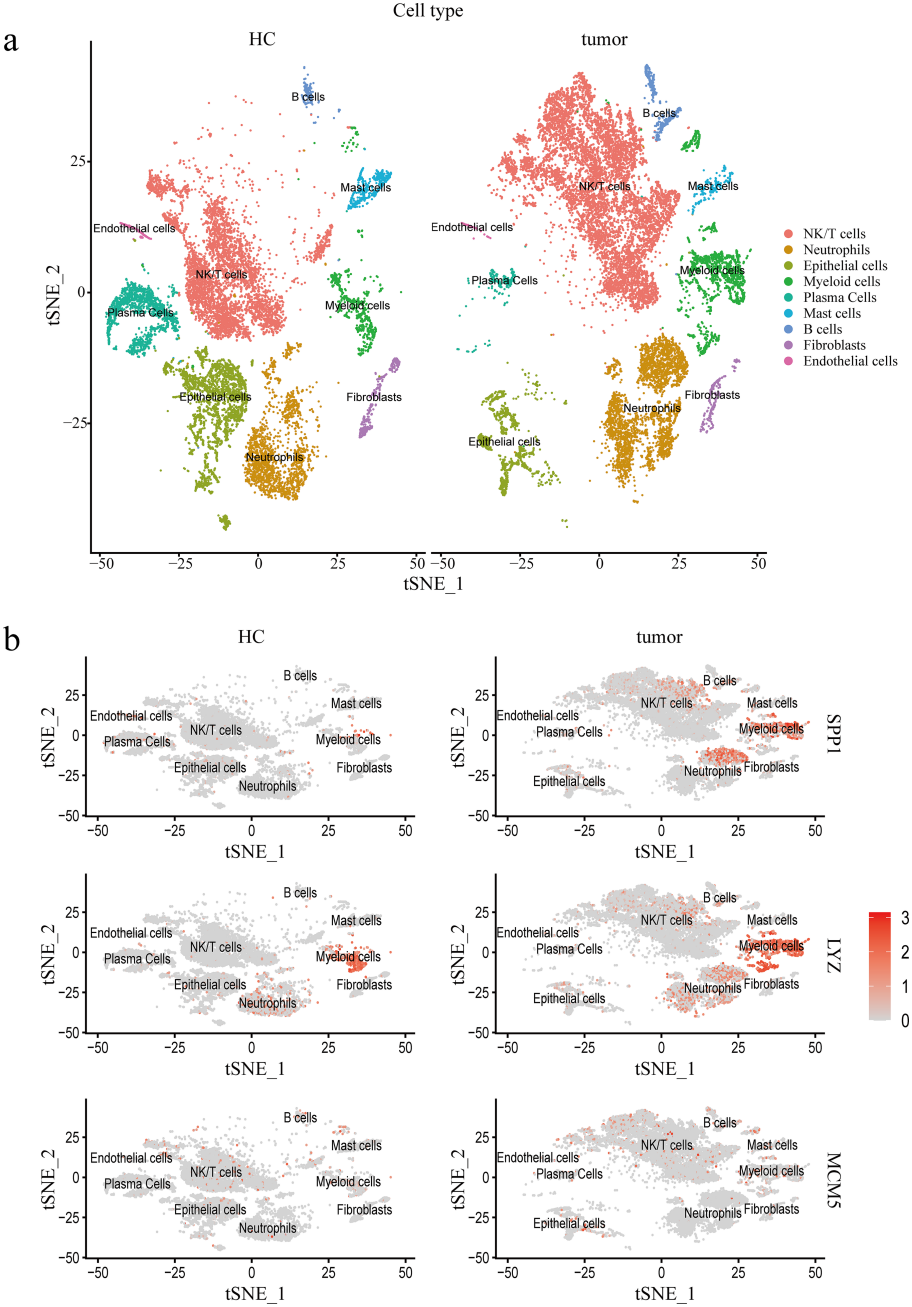
Single cell transcriptomic characterization of cervical cancer. **(a)** Single cells in the cervical cancer scRNA seq dataset are visualized using t-SNE by primary cell type. **(b)** The dot plot shows how three important core genes are expressed in various cell populations at varying rates. Healthy controls, HC; Single cell RNA Sequencing, scRNA-seq.

**Figure 8:**
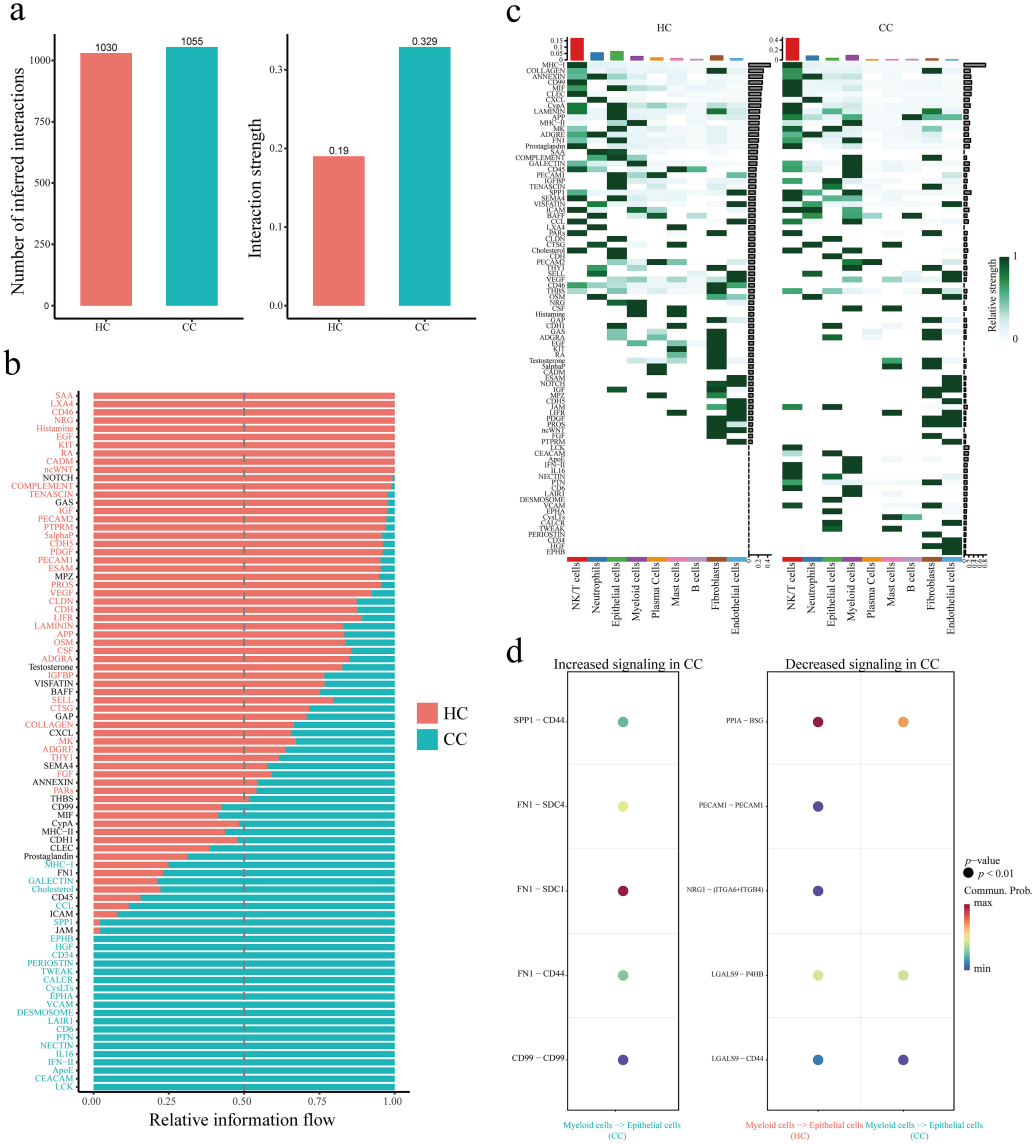
Single cell transcriptomic analysis of cellular communication in cervical cancer. **(a)** Comparison of the number and strength of inferred interactions between the control and cervical cancer groups. **(b)** Comparison of signal pathway enrichment between healthy control group and cervical cancer group. **(c)** Comparison of signaling pathway enrichment in each cell population between the healthy control group and the cervical cancer group. **(d)** Using Myeloid cells as ligands and Epithelial cells as receptors, compare the differences in ligand receptor pairs between the healthy control group and the cervical cancer group. Healthy controls, HC; Cervical cancer, CC; Single cell RNA Sequencing, scRNA-seq.

We analyzed the cervical cancer single cell transcriptome dataset (GSE208653) and divided the cervical cancer cell population into NK/T cells (Control group, 4,948 cells; cervical cancer group, 8,359 cells), Neutrophils (Control group, 1903 cells; cervical cancer group, 3,058 cells), Epithelial cells (Control group, 2,790 cells; cervical cancer group, 1,160 cells), Myeloid cells (Control group, 735 cells; cervical cancer group, 1701 cells), Plasma Cells (Control group, 1,565 cells; cervical cancer group, 242 cells), Mast cells (Control group, 484 cells; cervical cancer group, 184 cells), B cells (Control group, 162 cells; cervical cancer group, 446 cells), Fibroblasts (Control group, 333 cells; cervical cancer group, 231 cells), and Endothelial cells (Control group, 178 cells; cervical cancer group, 46 cells), a total of nine cell populations (Figure 7a). The expression of SPP1, LYZ and MCM5 in different cell populations was further evaluated. The tumor group exhibited statistically significant differences in SPP1, LYZ, and MCM5 expression levels in NK/T cells, myeloid cells, epithelial cells, and other cells compared to the healthy control group (Figure 7b; Supplementary Figures S5a–d). In comparison to the healthy control group, we observed that both the number and intensity of cell–cell interactions in the cervical cancer group were higher (Figure 8a). Subsequently, we compared the differences in signal pathway enrichment between the healthy control group and the cervical cancer disease group. The results showed that the communication intensity of signaling pathways such as LCK, CEACAM, IFN-II, IL16, NECTIN, PTN, LAIR1, VCAM, HGF, and SPP1 increased significantly in the cervical cancer group; the communication intensity of signaling pathways such as ncWNT, CADM, RA, KIT, EGF, Histamine, NRG, CD46, and LXA4 increased significantly in the healthy control group (Figure 8b). In addition, in terms of the overall signaling pathway intensity, SPP1 in the tumor group was more strongly expressed in NK/T cells, Neutrophils, and Myeloid cells (Figure 8c). On this basis, we used Myeloid cells as ligands and Epithelial cells as receptors to further analyze the differences in ligand receptor pairs between the healthy control group and the tumor group. The upregulated ligand receptor pairs in the cervical cancer group were SPP1 − CD44, FN1 − SDC4, FN1 − SDC1, FN1 − CD44, CD99 − CD99, and the downregulated ligand receptor pairs were PPIA – BSG, LGALS9 − P4HB, LGALS9 − CD44 (Figure 8d).

### 3.7 Differential expressions of SPP1, LYZ, and MCM5 in patients with rheumatoid arthritis combined with cervical cancer

**Figure 9:**
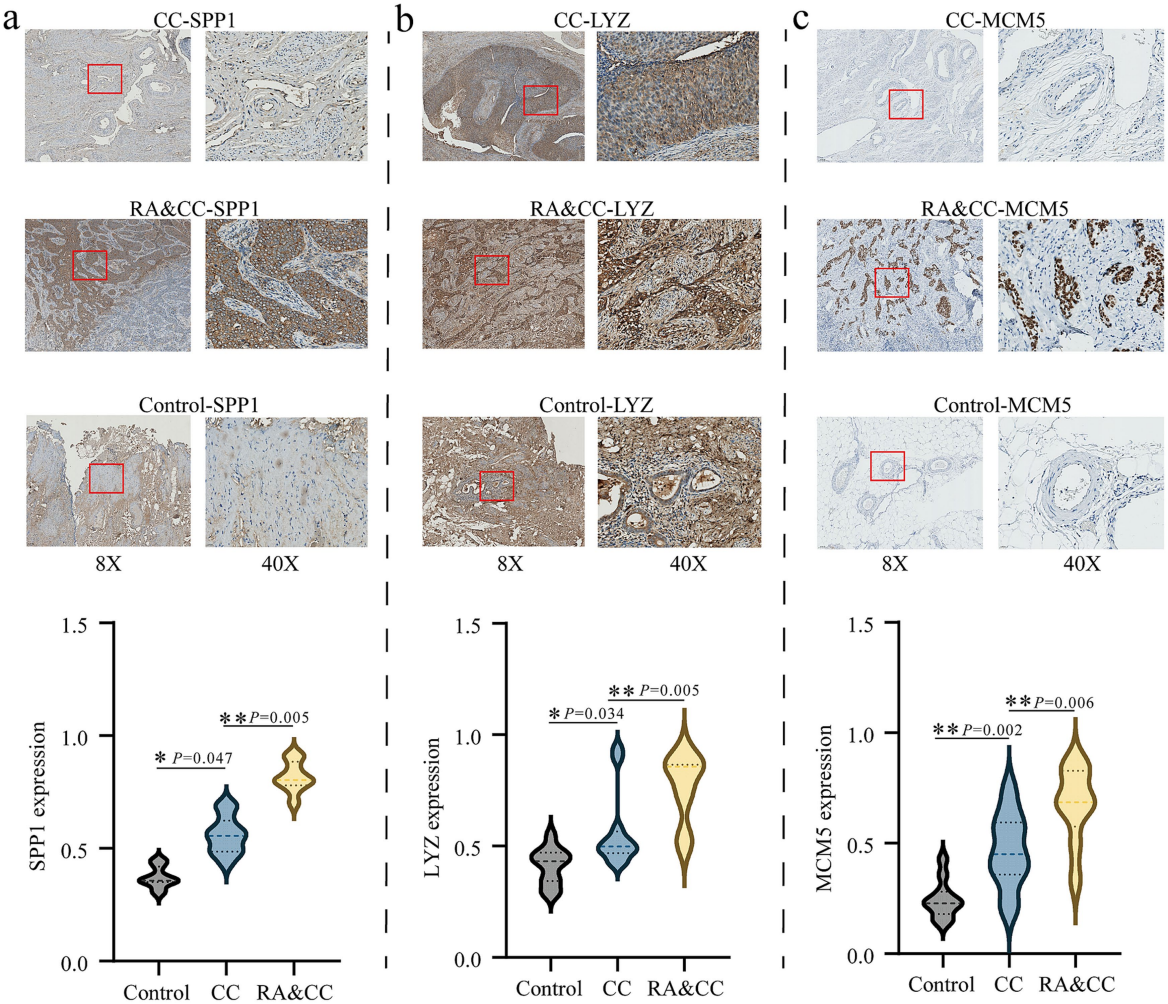
Immunohistochemistry analysis of the differential expression of SPP1, LYZ, and MCM5 in rheumatoid arthritis combined with cervical cancer patients and cervical cancer patients. **(a)** Analysis of SPP1 expression in the groups with cervical cancer, rheumatoid arthritis combined with cervical cancer, and healthy control group. **(b)** Analysis of LYZ expression in the groups with cervical cancer, rheumatoid arthritis combined with cervical cancer, and healthy control. **(c)** Analysis of MCM5 expression in the groups with cervical cancer, rheumatoid arthritis combined with cervical cancer, and healthy control. *p < 0.05, **p < 0.01. Healthy controls, HC; Rheumatoid arthritis, RA; Cervical cancer, CC.

To further verify the above bioinformatics results, we collected healthy controls, cervical cancer and RA combined with cervical cancer patients, and used immunohistochemistry to deeply analyze the distribution and expression of SPP1, LYZ and MCM5 in cervical tissues. The findings showed that cervical cancer tissues had increased expression levels of SPP1 (*p* = 0.047), LYZ (*p* = 0.034), and MCM5 (*p* = 0.002) in comparison to the healthy control group; this difference was statistically significant (Figure 9). Compared with cervical cancer patients, SPP1 (*p* = 0.005), LYZ (*p* = 0.005) and MCM5 (*p* = 0.006) were also highly expressed in RA combined with cervical cancer, and the difference was statistically significant (Figures 9a–c).

## 4 Discussion

Many research results show that autoimmune diseases show a higher susceptibility to cancer than the general population (30–33). Previous epidemiological data show that the overall risk of cervical cancer in RA patients is about 20% higher than that in the general population (34, 35). The occurrence and development of cervical cancer originates from a multi-level network composed of persistent HPV infection, destruction of host genome stability, chronic inflammation of the local cervix, and immune escape (36, 37). This multi-dimensional system jointly promotes the malignant transformation of cervical epithelium by activating carcinogenic signaling pathways. At the same time, as a multi-factor-mediated autoimmune disease, RA’s core pathogenesis is closely related to the persistent chronic inflammatory microenvironment. This inflammatory state may enhance HPV infection through multiple pathways, thereby increasing the susceptibility to cervical cancer transformation (8, 38). Specifically, gastrointestinal and vaginal dysbiosis can be triggered by various genetic and environmental factors, such as HPV infection, and engages in a bidirectional interaction with the host immune system, thereby promoting chronic inflammatory diseases. In RA, chronic synovial and systemic inflammation serves as a core mechanism driving disease progression, involving the activation of signaling pathways such as nuclear factor-κB (NF-κB), JAK–STAT, and MAPK, as well as the engagement of inflammasomes and the cGAS–STING pathway. This process promotes the activation of pro inflammatory T cell subsets such as Th1, Th9, and Th17, while suppressing the function of anti-inflammatory immune cells like Th2, Treg, and Breg cells (39, 40). Concurrently, dendritic cells, monocytes/macrophages, and neutrophils secrete large quantities of pro-inflammatory factors, further activating resident joint cells such as fibroblasts, chondrocytes, and osteoclasts, ultimately forming a self-reinforcing pro-inflammatory microenvironment. In the context of HPV induced cervical carcinogenesis and precancerous lesions, the viral oncoproteins E6 and E7 drive chronic inflammation by upregulating the COX-2/PGE2 pathway and other inflammatory mediators such as ROS, RNS, and PTGER. Within the tumor microenvironment (TME), the early stages are characterized by a predominance of M1 macrophages, NK cells, and CD4+/CD8 + T cells, which secrete anti-tumor factors including IL-2, IL-12, IL-18, and IFN-γ (41, 42). In advanced stages, however, the balance shifts toward immunosuppressive populations such as M2 macrophages, Th2/Th17 cells, Tregs, and Bregs, which suppress immune responses through the secretion of factors like IL-4, IL-10, IL-17, and TGF-β, thereby facilitating tumor immune escape. Throughout this process, sustained high levels of pro-inflammatory cytokines such as IL-6, IL-1β, and TNF-α maintain a state of chronic inflammation and drive disease progression. Consequently, a deeper understanding of the mechanisms linking chronic inflammation to carcinogenesis is of paramount importance for developing interventions aimed at preventing both autoimmune disorders and cancer (43).

Through the integration and analysis of single cell transcriptome data from RA and cervical cancer, this study methodically analyzed the particular signaling pathway regulation network of cervical cancer in the context of chronic inflammation. First, we analyzed the differentially expressed genes in the transcriptome data of cervical cancer patients (|log2FC| > 1, adj. *p* value < 0.05). Two thousand one hundred and seventy-two genes were lowly expressed in cervical cancer, 2,600 genes were highly expressed in cervical cancer, 493 genes were upregulated in RA patients, and 216 genes were downregulated. Subsequently, univariate and multivariate COX ratio analysis and LASSO regression were used to further screen genes related to the prognosis of cervical cancer, and a cervical cancer prognosis prediction model was constructed. On this basis, we successfully identified three hub genes (SPP1, LYZ, and MCM5) that were differentially expressed in the prognosis of RA and cervical cancer. Subsequently, molecular docking was used to predict the binding sites of hub genes and HPV 16 E6/E7. The analysis based on single cell transcriptome data revealed that the SPP1 signaling pathway was significantly enhanced in cervical cancer patients and was Myeloid cells and epithelial cells; lastly, it was found that the expression levels of SPP1, LYZ, and MCM5 were significantly higher in clinical tissue samples from patients with RA combined with cervical cancer when analyzed immunohistochemically.

SPP1, also known as osteopontin, often promotes cell invasion through integrin (αvβ3, CD44) signals, and can also enhance Th1/Th17 differentiation and inhibit Treg function (44). In tumor diseases, SPP1 can activate PI3K/AKT and FAK pathways, drive epithelial mesenchymal transition, induce MDSC proliferation, and inhibit CD8 + T cell activity (45, 46); in autoimmune diseases, SPP1 leads to local inflammatory cell infiltration (47, 48). Previous studies have found that in RA patients (48), SPP1 can interact with IFN-γ, enhance Th1 inflammatory response, and inhibit immune tolerance; synergize with RANKL, promote osteoclast differentiation and lead to bone erosion. The biological function of LYZ is mainly to hydrolyze bacterial cell wall peptidoglycan and participate in innate immune defense (49, 50); regulate macrophage polarization (M1/M2) and release of inflammatory factors. At present, the mechanism of LYZ in tumors is still unclear. LYZ activates macrophages to kill tumors and plays a role in tumor suppression; however, under chronic inflammation, LYZ releases proinflammatory factors (IL-6, TNF-α) to promote immunosuppression in the tumor microenvironment. In RA patients, synovial fluid LYZ levels are elevated, activating the NF-κB pathway to aggravate joint damage (50, 51). Excessive release of LYZ can also lead to degradation of healthy tissues, such as intestinal epithelial barrier damage in inflammatory bowel disease (52, 53). MCM5 is a core component of the DNA replication initiation complex, ensuring stable genome replication, regulating G1/S phase transition, and driving cell proliferation. MCM5 is associated with genomic instability, and its high expression can lead to abnormal proliferation of cervical cancer, liver cancer (54–57). Previous studies have found that when B/T cells are overactivated, MCM5 related lymphocyte infiltration is often observed. However, there is no direct evidence demonstrating a clear interactive relationship among SPP1, LYZ, and MCM5. We speculate that B2M might act as a common interacting protein for SPP1, LYZ, and MCM5. B2M is the light chain component of MHC class I molecules and is crucial for the stability and antigen presenting function of MHC class I (58, 59). By participating in the antigen presentation process of MHC class I molecules, B2M influences T cell recognition and killing of infected or tumor cells. This could represent another potential mechanism by which SPP1, LYZ, and MCM5, in addition to their individual functions, collectively regulate chronic inflammation through their interactions, thereby increasing the risk of cervical carcinogenesis.

The study merged the transcriptome data of prior RA and cervical cancer patients with the cohort of RA combined with cervical cancer we collected for thorough analysis, and found that SPP1, LYZ, and MCM5 are proteins closely related to the HPV 16 virus and may be candidate biomarkers for patients with RA and cervical cancer. At the same time, based on the level of single cell sequencing, we further found that SPP1 is an important signaling pathway connecting Myeloid cells and epithelial cells. According to previous studies, a number of inflammatory and autoimmune conditions, such as Crohn’s disease, cirrhosis, obesity, atherosclerosis, cancer, multiple sclerosis (MS), RA, and osteoarthritis (OA), have elevated SPP1 concentrations (40, 60–62). In the immunopathological process of RA, immune cells and cytokines infiltrate synovial tissue. The integrin receptors and their ligands are upregulated in this condition, which leads to the activation of fibroblast like synoviocytes (FLS), heightened production of matrix metalloproteinases (MMPs) and cytokines, and aggravated cell extravasation (63, 64). The above series of changes will trigger the invasion and degradation of cartilage, thereby producing extracellular matrix (ECM) fragments. Integrins might be further activated by these ECM fragments. Increased αvβ3 integrin expression in synovial tissue in RA disease encourages FLS invasion and attachment to the cartilage pannus junction, which triggers the release of MMP and cathepsin and ultimately results in joint destruction. Tumor associated macrophages (TAMs) (65, 66) are the most common immune cells in the TME, and play a vital role in the occurrence, development and metastasis of cancer (67, 68). SPP1 can promote the interaction between cancer cells and macrophages through a variety of complex mechanisms, thereby enhancing the proliferation, invasion and migration of cancer cells. Specifically, SPP1, as a potent chemokine for macrophages, can recruit TAMs from peripheral blood monocytes to the tumor microenvironment and promote M2 like activation of TAMs (69, 70) in this environment. When SPP1 is blocked, glioma cells’ capacity to attract macrophages is greatly diminished. By activating the colony stimulating factor 1 receptor (CSF1R) pathway in macrophages, tumor derived SPP1 can also increase the expression of programmed death ligand 1 (PD-L1) and cause macrophage reprogramming to M2 type through the integrin and protein tyrosine kinase 2 (PTK2)-Akt signaling pathways (41, 71). In addition, SPP1 increases the expression of cyclooxygenase 2 (COX-2) in macrophages by interacting with α9β1 integrin on macrophage receptors, which activates the p38 and extracellular signal regulated kinase (ERK) signaling pathways (72). COX 2 derived prostaglandin E2 (PGE2) and matrix metalloproteinase 9 (MMP9) can stimulate angiogenesis and further expand the degree of macrophage activation. At the same time, the latest studies have shown that SPP1 + TAM, as a new macrophage subset, presents a higher immune infiltration rate and has the characteristics of promoting tumor development. Increased SPP1 expression in TAMs can cause T cell inhibition and inhibit CD8 + T cell production of interferon-γ (IFN-γ) while also upregulating PD-L1 expression in cancer cells via the NF-κB pathway. This effect might be connected to SPP1/CD44 activation (73–75) signaling pathway, which in turn promotes tumor immune tolerance and immune escape, and enhances the resistance of tumor cells to anti-tumor immunotherapy. The findings suggest that chronic inflammation is a prominent characteristic in patients with RA and cervical cancer. Additionally, SPP1 plays a role in mediating interactions between macrophages and epithelial cells by regulating inflammation, epithelial-mesenchymal transition (EMT) (76), immune evasion, and metabolism, thereby contributing to tumor progression. Combined intervention of these molecules may provide a novel treatment option for patients with RA combined with cervical cancer. It is evident that SPP1 exhibits significant associations with multiple factors within the TME. By binding to receptors such as CD44 and integrins, SPP1 activates key signaling pathways including PI3K/AKT/mTOR, Slug/Snail, MAPK/NF-κB, and Ras/Raf/ERK, thereby promoting tumor cell proliferation, migration, invasion, adhesion, and EMT. Studies have confirmed that BET inhibitors and cytarabine can suppress SPP1 transcription, while Entrectinib directly binds to SPP1 and reduces its expression (77–79), resulting in synergistic anti-tumor effects. It should be noted, however, that SPP1 plays a crucial role in bone metabolism homeostasis, and both excessive inhibition and activation may lead to bone abnormalities. Future research should focus on developing precise SPP1 targeted therapeutic strategies, such as highly specific antibodies, RNA interference techniques, or small molecule modulators, to achieve controllable regulation of SPP1 activity. Through tissue specific expression modulation, it is expected to enhance therapeutic efficacy while minimizing systemic adverse effects.

Even though our study showed that SPP1, LYZ, and MCM5 have good clinical value and diagnostic performance in patients with cervical cancer and RA, there are still certain limitations. First, the dataset has a small sample size. Future studies should focus on collecting patients with RA and cervical cancer for high throughput sequencing to further reveal the role of SPP1, LYZ, and MCM5 in the disease. In addition, the number of clinical cohorts of patients with RA combined with cervical cancer we collected is limited, and the sample size will be expanded for further verification. Additionally, immunosuppressive therapies commonly used in RA may influence HPV persistence and the expression of the identified biomarkers, potentially confounding the observed associations. Future studies should account for treatment related variables to clarify these relationships. In conclusion, these results require additional *in vitro* and *in vivo* testing to confirm them.

## 5 Conclusion

This study explored the molecular mechanism of comorbidity between RA and cervical cancer by integrating transcriptomic analysis and validating it in individuals with cervical cancer combined with RA, and successfully identified three key hub genes (SPP1, LYZ, and MCM5), which were significantly upregulated in both RA and cervical cancer. The results showed that SPP1, LYZ, and MCM5 not only had high diagnostic potential in patients with RA and cervical cancer but also showed specific binding sites with HPV 16 E6/E7 proteins through molecular docking simulation. Single cell sequencing data showed that SPP1 was an important pathway for regulating the immune microenvironment and epithelial cells. Despite the limitations of limited sample size, this study provides an important paradigm for the exploration of the mechanism of chronic inflammation related cancers, and the combined intervention of these molecules may provide a new treatment idea for patients with RA combined with cervical cancer.

## Data Availability

Publicly available datasets were analyzed in this study. This data can be found here: GEO database: https://www.ncbi.nlm.nih.gov/geo/, TCGA database: https://portal.gdc.cancer.gov/, and GTEx database: https://gtexportal.org/home/.

## Footnotes

^1^ https://portal.gdc.cancer.gov/

^2^ https://cn.string-db.org/
