# Supplementary material for: SPP1, LYZ, and MCM5: potential diagnostic biomarkers for rheumatoid arthritis and cervical cancer comorbidity
Source: Front Med (Lausanne). 2025 Nov 26;12:1693787. doi: 10.3389/fmed.2025.1693787 (PMC12689957; doi:10.3389/fmed.2025.1693787)
Supplement: Supplementary file 1 [file Data_Sheet_1.docx]

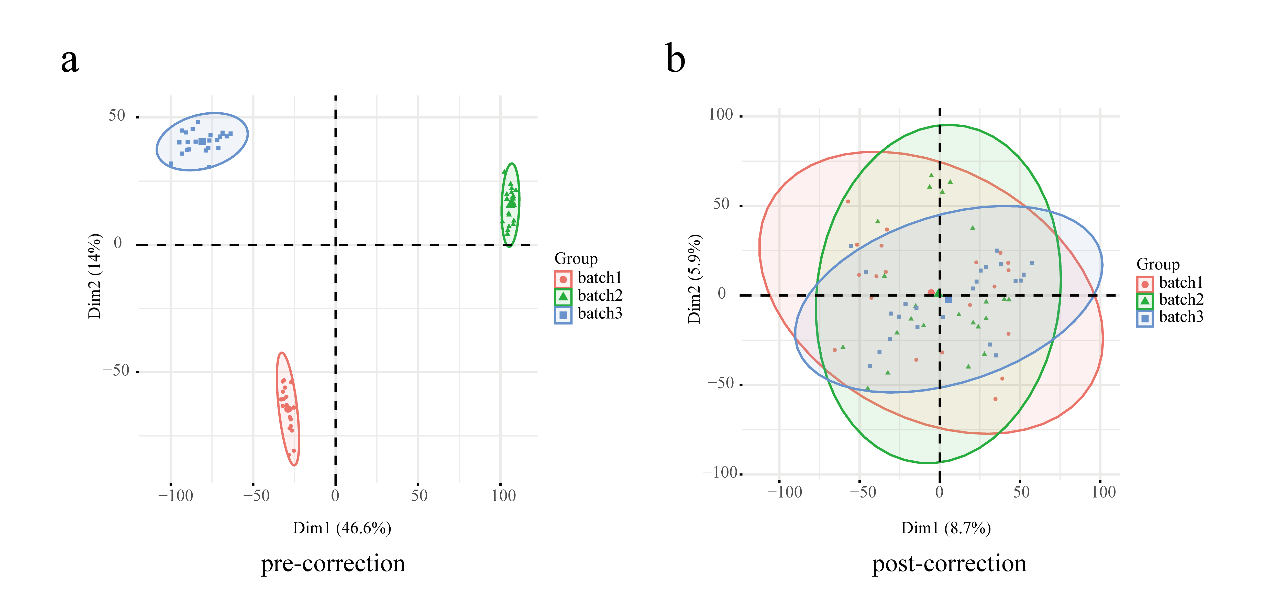


Figure S1: Evaluation of batch effect correction by Principal Component Analysis (PCA).​​ (a) Before correction, samples from different batches were clearly separated, indicating a significant batch effect. (b) After correction, the samples are more evenly distributed, suggesting that the batch effects have been effectively mitigated.


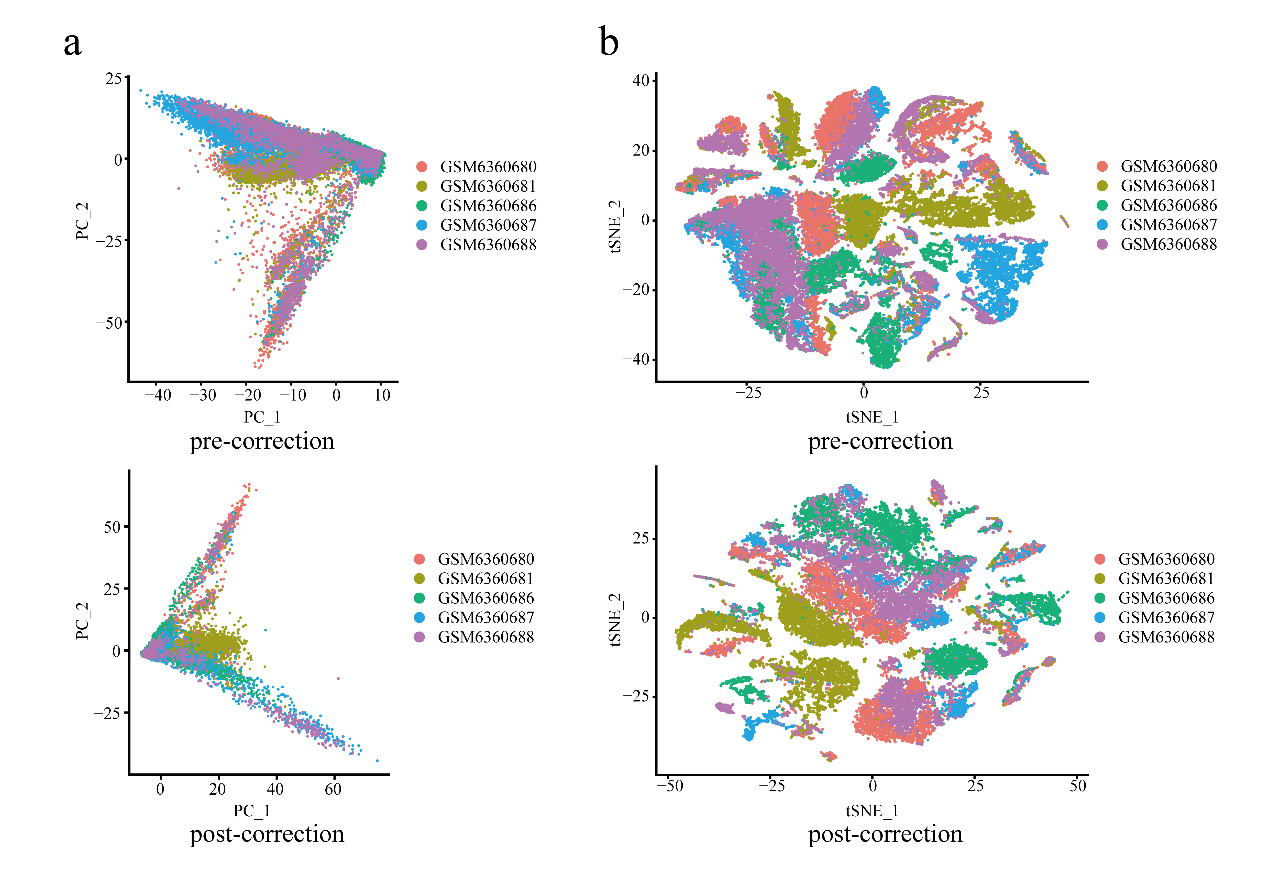


Figure S2: Evaluation of batch effect correction by Principal Component Analysis (PCA) and t-distributed stochastic neighbor embedding (t-SNE).​​ ​​ (a) PCA shows the distribution of samples in multidimensional space before and after Harmony removes batch effects. (b) t-SNE shows the distribution of samples in multidimensional space before and after Harmony removes batch effects.


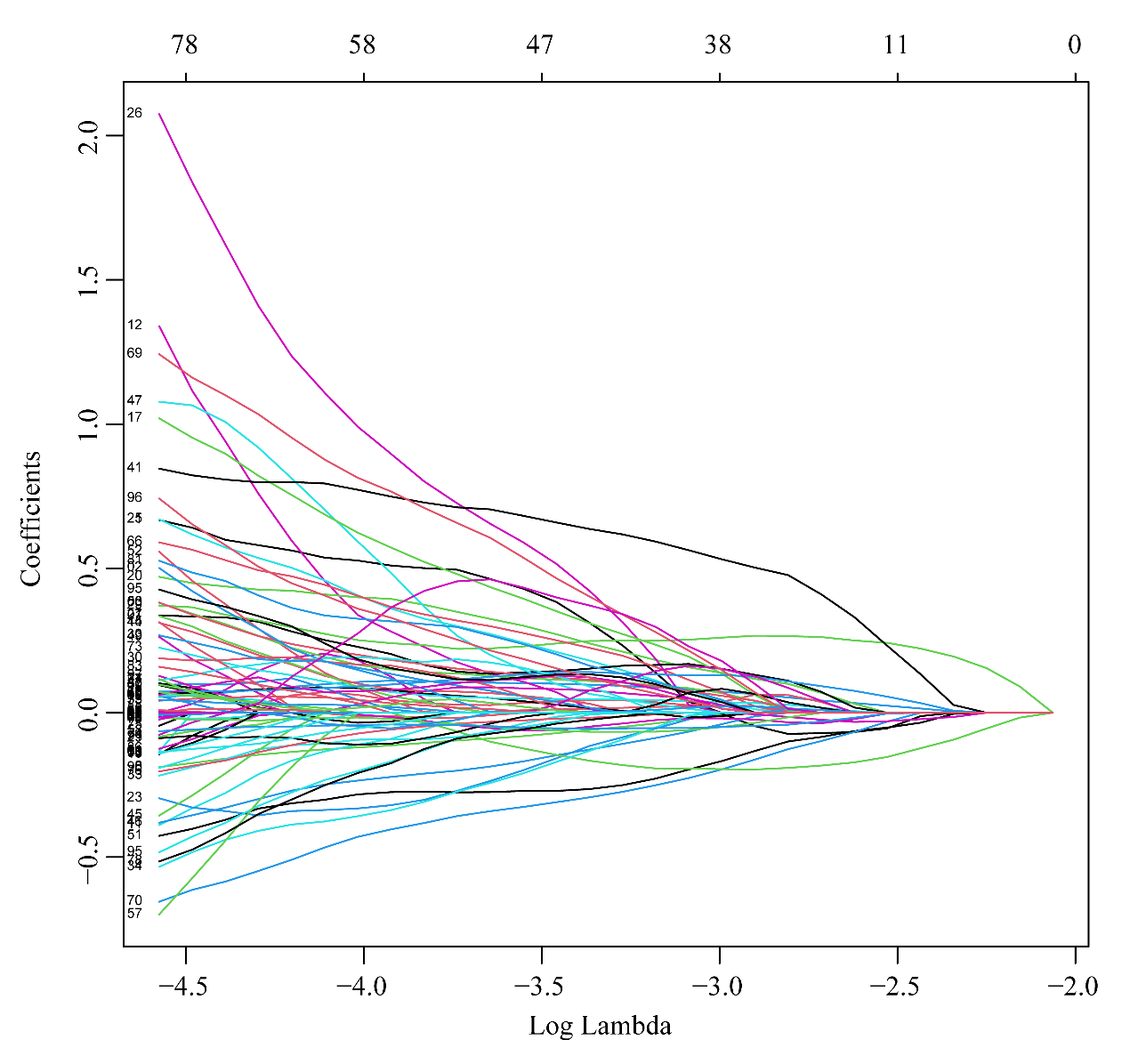


Figure S3: LASSO regression analysis of genes associated with cervical cancer prognosis.


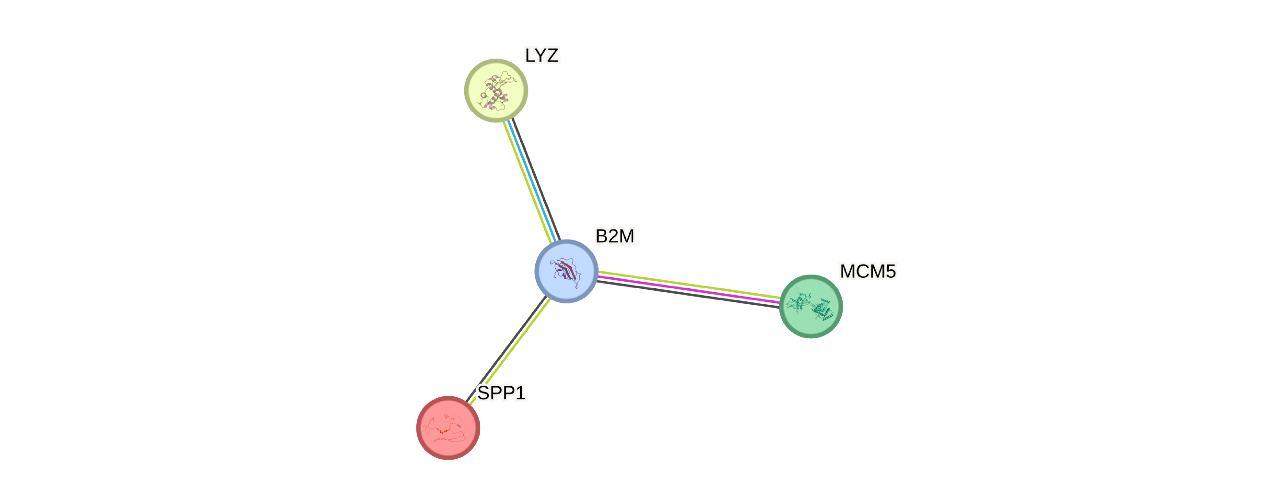


Figure S4: Prediction of a shared interaction network among SPP1, LYZ, and MCM5.​​ The protein-protein interaction (PPI) network was constructed using the STRING database (https://string-db.org/).


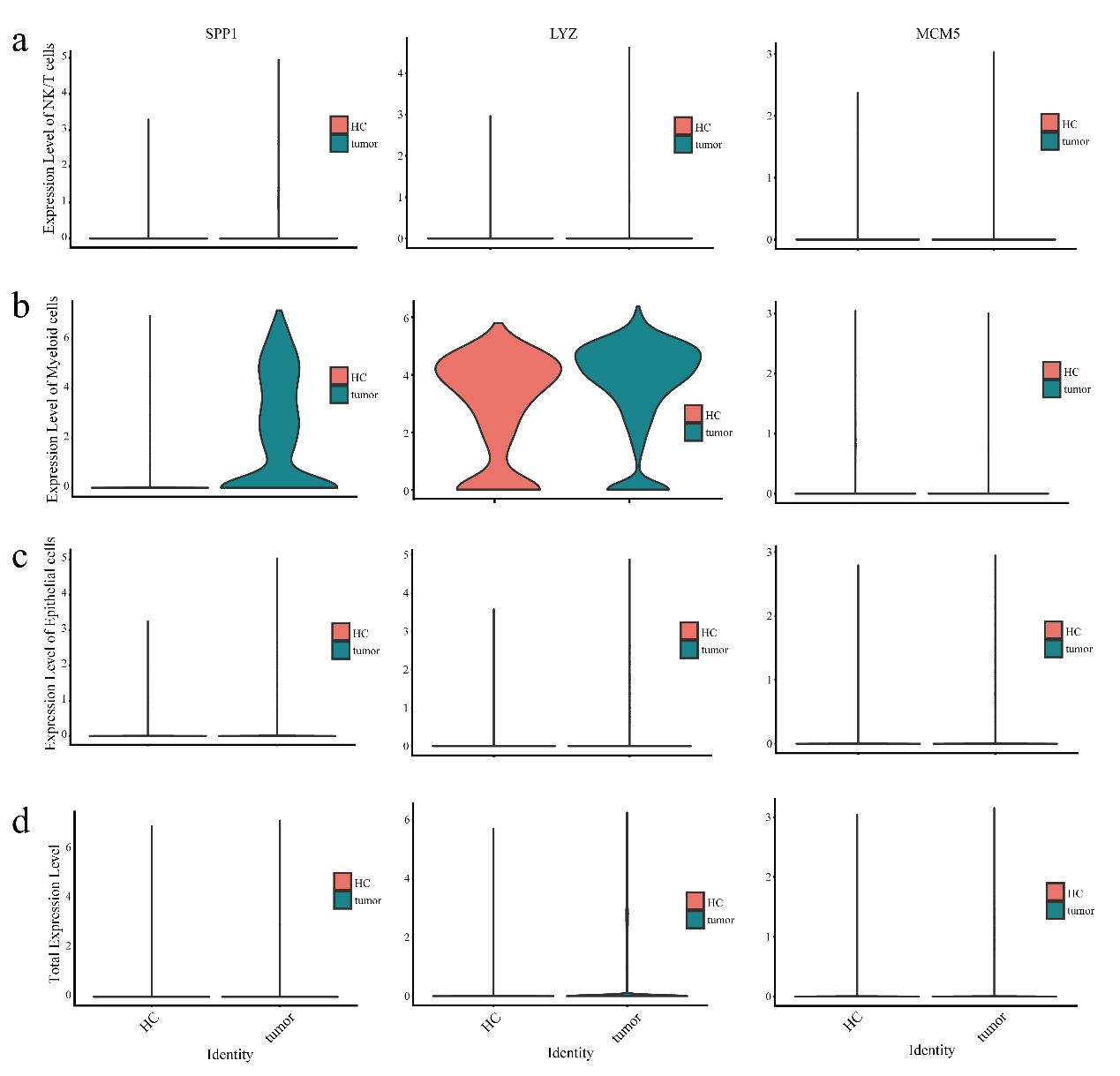


Figure S5: Violin plot comparing the expression of three essential core genes in cervical cancer to controls (a) Violin plots show the expression of SPP1, LYZ and MCM5 in NK/T cells in the control group and the cervical cancer group. (b) Violin plots show the expression of SPP1, LYZ and MCM5 in myeloid cells in the control group and the cervical cancer group. (c) Violin plots show the expression of SPP1, LYZ and MCM5 in epithelial cells in the control group and the cervical cancer group. (d) Violin plots show the expression of SPP1, LYZ and MCM5 in overall cell populations in the control group and the cervical cancer group.

Table S1: Clinical characteristics of patients in the three groups.

|  | Control | Cervical cancer | Rheumatoid arthritis combined with cervical cancer |
| --- | --- | --- | --- |
| Age | 54.27 ± 8.27 | 55.08 ± 8.11 | 56.89 ± 6.37 |
| Disease duration years (Cervical cancer) | - | 1.8 ± 0.71 | 2.17± 1.17 |
| Disease duration years (Rheumatoid arthritis) | - | - | 6.89 ± 3.86 |
| Treatment history（NSAIDs） | - | - | 9 (100.0) |
| treatment history（MTX/LEF） | - | - | 4 (44.4) |
| Stopping medication of time (≥ 1 year) | - | - | 4 (44.4) |
| HPV infection status | - | 9 (100.0) | 9 (100.0) |

Table S2: Screening of prognostic genes related to cervical cancer using univariate cox proportional model.

| Id | HR | HR.95L | HR.95H | *P* value |
| --- | --- | --- | --- | --- |
| NPHP3 | 2.202395 | 1.015011 | 4.778806 | 0.045753 |
| MT1A | 1.302963 | 1.088186 | 1.560131 | 0.003983 |
| HLA-DRB1 | 0.862358 | 0.756223 | 0.983389 | 0.027109 |
| MIR100HG | 1.597944 | 1.154456 | 2.2118 | 0.004715 |
| IGHGP | 0.874695 | 0.781819 | 0.978604 | 0.019408 |
| IGLC3 | 0.89866 | 0.818771 | 0.986343 | 0.024483 |
| RMI2 | 0.698964 | 0.518929 | 0.94146 | 0.018426 |
| LYZ | 0.861188 | 0.751427 | 0.986982 | 0.031687 |
| OSBPL5 | 1.502641 | 1.050351 | 2.149691 | 0.025825 |
| CXCL8 | 1.275897 | 1.13168 | 1.438493 | 6.85E-05 |
| CRIP1 | 0.709775 | 0.52148 | 0.966058 | 0.029294 |
| SPNS1 | 2.820975 | 1.342541 | 5.927491 | 0.006191 |
| APEX2 | 0.455333 | 0.273105 | 0.759151 | 0.002557 |
| RBM4 | 2.442911 | 1.140977 | 5.230439 | 0.021477 |
| SLC25A5 | 0.702888 | 0.510002 | 0.968723 | 0.031231 |
| ENO1 | 1.463046 | 1.04386 | 2.050566 | 0.027163 |
| GLT8D2 | 1.463066 | 1.113099 | 1.923065 | 0.006369 |
| AC004967.1 | 1.832324 | 1.181976 | 2.840505 | 0.006781 |
| IGLV3-21 | 0.882641 | 0.79792 | 0.976357 | 0.015322 |
| PPP1R14A | 1.542036 | 1.202488 | 1.977463 | 0.000642 |
| FAM83H | 1.55677 | 1.102084 | 2.199046 | 0.012021 |
| CIRBP | 0.656588 | 0.432807 | 0.996074 | 0.047877 |
| POLR2J3 | 0.551711 | 0.306321 | 0.993679 | 0.047581 |
| DBNDD2 | 1.564994 | 1.051392 | 2.32949 | 0.027321 |
| CHMP4C | 1.822844 | 1.184713 | 2.804697 | 0.006315 |
| ADCY4 | 1.997554 | 1.108423 | 3.599908 | 0.021306 |
| MRPL38 | 2.00479 | 1.194125 | 3.365795 | 0.008511 |
| SERPINF1 | 0.769931 | 0.622111 | 0.952876 | 0.016228 |
| PEAR1 | 2.142413 | 1.594637 | 2.878355 | 4.25E-07 |
| RAP1B | 2.201602 | 1.178539 | 4.112761 | 0.013316 |
| LMBR1L | 1.924028 | 1.071907 | 3.45355 | 0.028335 |
| MAP7 | 1.742127 | 1.258325 | 2.411943 | 0.000825 |
| CHAF1B | 0.493828 | 0.308652 | 0.7901 | 0.003256 |
| AQP3 | 0.887047 | 0.798742 | 0.985113 | 0.025071 |
| FBN1 | 1.279309 | 1.010249 | 1.620027 | 0.040894 |
| ARHGEF25 | 1.397405 | 1.025248 | 1.904653 | 0.034194 |
| AL441992.1 | 0.63533 | 0.443542 | 0.910048 | 0.013359 |
| NDUFA11 | 0.534432 | 0.347628 | 0.82162 | 0.004299 |
| LAMA4 | 1.463406 | 1.10574 | 1.936765 | 0.007747 |
| CD3D | 0.75762 | 0.634237 | 0.905006 | 0.00221 |
| TXNDC5 | 1.819148 | 1.174579 | 2.817434 | 0.007343 |
| CBX7 | 0.556586 | 0.362362 | 0.854912 | 0.007454 |
| TRIOBP | 0.551314 | 0.324089 | 0.937849 | 0.028043 |
| ADAMTS9 | 1.464756 | 1.112467 | 1.928605 | 0.006542 |
| NPR2 | 1.49395 | 1.086205 | 2.054756 | 0.01357 |
| DES | 0.807701 | 0.670416 | 0.973098 | 0.024648 |
| ZNF280D | 2.809413 | 1.152598 | 6.847835 | 0.023063 |
| IGHV1-2 | 0.893978 | 0.803107 | 0.995131 | 0.040443 |
| EIF3C | 2.809468 | 1.373068 | 5.748521 | 0.004685 |
| CCL5 | 0.815521 | 0.706655 | 0.941159 | 0.005279 |
| RNASEH2A | 0.454861 | 0.301867 | 0.685395 | 0.000166 |
| FEZ1 | 1.300333 | 1.045892 | 1.616674 | 0.018087 |
| TMEM98 | 1.260108 | 1.027124 | 1.545941 | 0.026654 |
| ZNF667-AS1 | 0.716127 | 0.515342 | 0.995141 | 0.046704 |
| IGHM | 0.882063 | 0.803751 | 0.968006 | 0.008158 |
| ETV7 | 0.767384 | 0.627405 | 0.938593 | 0.009975 |
| ANLN | 1.582675 | 1.133495 | 2.209854 | 0.007024 |
| PCDHGC3 | 1.406139 | 1.106789 | 1.786454 | 0.00526 |
| NKG7 | 0.833241 | 0.714436 | 0.971803 | 0.020105 |
| PTPRM | 1.445072 | 1.117859 | 1.868065 | 0.004946 |
| GET4 | 2.422266 | 1.157452 | 5.069213 | 0.018872 |
| HIST1H4I | 0.701678 | 0.555563 | 0.886222 | 0.002941 |
| SPP1 | 1.19222 | 1.051558 | 1.351698 | 0.006055 |
| IGHG3 | 0.886625 | 0.808131 | 0.972744 | 0.010951 |
| ARMCX1 | 1.378843 | 1.091858 | 1.74126 | 0.006975 |
| CYTL1 | 1.568814 | 1.195559 | 2.058599 | 0.001161 |
| AC245595.1 | 2.637486 | 1.590636 | 4.373303 | 0.000171 |
| ANKRD22 | 0.7408 | 0.613763 | 0.894131 | 0.001773 |
| HK2 | 1.284102 | 1.014324 | 1.625634 | 0.037695 |
| RPL41P5 | 0.659454 | 0.517013 | 0.841137 | 0.000798 |
| KLHDC7B | 0.899863 | 0.809867 | 0.99986 | 0.049695 |
| SYCP2 | 0.830572 | 0.709772 | 0.971931 | 0.020613 |
| CA9 | 1.167218 | 1.042231 | 1.307193 | 0.007456 |
| AGRN | 1.384228 | 1.035771 | 1.849915 | 0.027985 |
| PCNA | 0.677214 | 0.474388 | 0.966761 | 0.031866 |
| FGFR3 | 0.874652 | 0.76551 | 0.999355 | 0.048901 |
| CDH3 | 1.224951 | 1.012606 | 1.481824 | 0.036713 |
| AC099850.3 | 1.431439 | 1.058663 | 1.935476 | 0.019788 |
| SERINC2 | 1.261357 | 1.008251 | 1.578002 | 0.042167 |
| RIBC2 | 0.547445 | 0.423214 | 0.708144 | 4.48E-06 |
| BAIAP2L1 | 1.736807 | 1.101782 | 2.737834 | 0.017436 |
| WSB1 | 1.722063 | 1.091581 | 2.716701 | 0.019455 |
| ILK | 1.938331 | 1.170724 | 3.209234 | 0.010091 |
| UBALD2 | 0.673141 | 0.466737 | 0.970823 | 0.034137 |
| IGHG2 | 0.898189 | 0.820728 | 0.982961 | 0.019624 |
| AC018738.1 | 0.760968 | 0.609164 | 0.950601 | 0.016118 |
| MFAP5 | 1.165595 | 1.006306 | 1.350098 | 0.040973 |
| UCP2 | 0.712965 | 0.570326 | 0.891279 | 0.002973 |
| HSPB1P1 | 0.802162 | 0.677286 | 0.950062 | 0.01067 |
| MMP3 | 1.182798 | 1.052192 | 1.329617 | 0.004921 |
| ZIC2 | 0.783789 | 0.646754 | 0.94986 | 0.012969 |
| MEST | 1.295451 | 1.027779 | 1.632836 | 0.028381 |
| GATD3B | 3.157061 | 1.582352 | 6.29887 | 0.001106 |
| TSC22D3 | 0.730495 | 0.583123 | 0.915112 | 0.006303 |
| CHAF1A | 0.470986 | 0.304537 | 0.728411 | 0.000713 |
| GSN | 0.713492 | 0.515103 | 0.98829 | 0.042273 |
| SMC1B | 0.787197 | 0.644055 | 0.962152 | 0.019451 |
| HENMT1 | 0.537922 | 0.398824 | 0.725535 | 4.87E-05 |
| PCSK7 | 2.17575 | 1.181812 | 4.005616 | 0.012546 |
| AC103810.3 | 1.387459 | 1.079476 | 1.783312 | 0.010554 |
| ST5 | 1.404362 | 1.007594 | 1.957368 | 0.045003 |
| HOXA10 | 1.413502 | 1.086434 | 1.839032 | 0.009956 |
| ZCCHC24 | 1.518028 | 1.059391 | 2.175222 | 0.022947 |
| TRPV4 | 0.775573 | 0.610022 | 0.986054 | 0.038023 |
| MPRIP | 1.572322 | 1.033094 | 2.393003 | 0.034695 |
| GINS2 | 0.653181 | 0.43929 | 0.971213 | 0.035354 |
| CKAP2 | 1.509945 | 1.008026 | 2.261781 | 0.045637 |
| CXCL1 | 1.143639 | 1.024153 | 1.277065 | 0.017132 |
| HLA-DRA | 0.836166 | 0.727268 | 0.96137 | 0.011959 |
| SCD | 1.229174 | 1.024255 | 1.47509 | 0.026588 |
| ALG1L | 0.774624 | 0.647724 | 0.926387 | 0.005148 |
| DEPP1 | 1.239812 | 1.003006 | 1.532526 | 0.046843 |
| TSPYL2 | 0.527066 | 0.320561 | 0.866603 | 0.011593 |
| WFDC21P | 0.861066 | 0.766384 | 0.967445 | 0.011841 |
| CLEC3B | 0.580169 | 0.37486 | 0.897927 | 0.014561 |
| AP002956.1 | 1.388066 | 1.125733 | 1.711532 | 0.002154 |
| SKA3 | 1.787371 | 1.08273 | 2.950593 | 0.023162 |
| COL5A1 | 1.18782 | 1.016151 | 1.388491 | 0.030685 |
| TFRC | 1.362962 | 1.127995 | 1.646873 | 0.001339 |
| NOMO2 | 1.950913 | 1.178928 | 3.228409 | 0.009309 |
| WDR86 | 1.640303 | 1.038602 | 2.590593 | 0.033804 |
| MIR621 | 1.445673 | 1.070643 | 1.95207 | 0.016153 |
| MAN1C1 | 0.577394 | 0.356733 | 0.93455 | 0.025386 |
| SNRPN | 0.788344 | 0.634669 | 0.97923 | 0.03158 |
| MEG3 | 1.512476 | 1.016382 | 2.250711 | 0.041342 |
| DIXDC1 | 2.012134 | 1.15047 | 3.519156 | 0.01423 |
| RUFY3 | 1.871566 | 1.050394 | 3.334708 | 0.033437 |
| TRBV28 | 0.711258 | 0.584449 | 0.865581 | 0.000672 |
| HIST1H3H | 0.694269 | 0.536228 | 0.898889 | 0.005626 |
| HSPG2 | 1.36591 | 1.076337 | 1.733387 | 0.010314 |
| GTF2I | 1.65118 | 1.128407 | 2.416145 | 0.009824 |
| FOXE1 | 0.853971 | 0.73104 | 0.997573 | 0.046525 |
| RPL17 | 1.737616 | 1.100301 | 2.744075 | 0.01779 |
| RGS5 | 0.735606 | 0.561868 | 0.963067 | 0.025502 |
| PRPF40B | 2.673904 | 1.423568 | 5.022421 | 0.002228 |
| MEIS2 | 0.600401 | 0.375559 | 0.959852 | 0.033078 |
| UBE2V1 | 3.075695 | 1.603308 | 5.900237 | 0.000724 |
| PTGDS | 0.823037 | 0.711272 | 0.952364 | 0.008911 |
| CLK3 | 2.664653 | 1.230345 | 5.771047 | 0.012929 |
| RASGRP2 | 0.531159 | 0.288655 | 0.977395 | 0.042007 |
| CCZ1 | 4.44596 | 2.202477 | 8.974694 | 3.14E-05 |
| ACKR1 | 0.770038 | 0.623271 | 0.951366 | 0.015432 |
| MCM2 | 0.723399 | 0.531676 | 0.984256 | 0.039306 |
| HIST1H1C | 0.75667 | 0.641179 | 0.892963 | 0.000968 |
| CDC45 | 0.646845 | 0.426571 | 0.980863 | 0.040274 |
| NDRG2 | 0.75002 | 0.602968 | 0.932935 | 0.009783 |
| FUNDC2 | 0.57794 | 0.343907 | 0.971235 | 0.038437 |
| GGT6 | 0.813414 | 0.675218 | 0.979893 | 0.029723 |
| DAAM2 | 1.811454 | 1.249481 | 2.626183 | 0.001717 |
| TGFA | 1.397047 | 1.098407 | 1.776884 | 0.006432 |
| RPL41P2 | 0.67412 | 0.467606 | 0.971841 | 0.0346 |
| PDE2A | 1.946508 | 1.284934 | 2.948706 | 0.001672 |
| CENPM | 0.515739 | 0.36771 | 0.72336 | 0.000125 |
| ASF1B | 0.580235 | 0.41473 | 0.811788 | 0.001488 |
| PLIN2 | 1.271152 | 1.032213 | 1.5654 | 0.02392 |
| FRAT2 | 0.659382 | 0.45194 | 0.962039 | 0.030714 |
| PHLDB1 | 1.616858 | 1.106245 | 2.363156 | 0.013086 |
| APOBEC3B | 0.745009 | 0.639 | 0.868604 | 0.000171 |
| DEF6 | 0.606814 | 0.442841 | 0.831502 | 0.001883 |
| SLC7A5 | 1.168284 | 1.010479 | 1.350734 | 0.035662 |
| C1QTNF1 | 1.279693 | 1.019487 | 1.606314 | 0.033473 |
| CMAHP | 0.776232 | 0.615895 | 0.97831 | 0.031895 |
| CCL20 | 1.157004 | 1.027125 | 1.303307 | 0.016372 |
| DSG2 | 1.539038 | 1.180415 | 2.006614 | 0.001446 |
| DLG4 | 1.60249 | 1.092153 | 2.351294 | 0.015927 |
| MPDZ | 1.718346 | 1.209464 | 2.44134 | 0.002517 |
| WDR27 | 2.337887 | 1.402876 | 3.896079 | 0.001118 |
| TNFRSF12A | 1.368031 | 1.104944 | 1.693759 | 0.004031 |
| EEF1D | 1.644815 | 1.023624 | 2.642978 | 0.039739 |
| LINC01089 | 0.50019 | 0.313488 | 0.798084 | 0.00366 |
| APOD | 0.80339 | 0.648406 | 0.995417 | 0.045288 |
| LINC01133 | 0.861416 | 0.753373 | 0.984953 | 0.029132 |
| TFPI | 1.480727 | 1.204626 | 1.820111 | 0.000193 |
| PDLIM7 | 1.385626 | 1.038179 | 1.849353 | 0.026805 |
| SMTN | 1.340086 | 1.007062 | 1.783238 | 0.044618 |
| PLAU | 1.198398 | 1.007977 | 1.424792 | 0.040369 |
| TMEM255B | 2.21467 | 1.351121 | 3.630144 | 0.001613 |
| MCM5 | 0.514122 | 0.353431 | 0.747874 | 0.000503 |
| MIR9-3HG | 0.744449 | 0.618453 | 0.896114 | 0.001812 |
| GABRP | 0.906936 | 0.828903 | 0.992315 | 0.033334 |
| OSR2 | 0.706074 | 0.523416 | 0.952473 | 0.02268 |
| GALNT3 | 1.476433 | 1.116537 | 1.952335 | 0.006272 |
| SUSD4 | 0.820556 | 0.706747 | 0.952692 | 0.009428 |
| E2F1 | 0.675235 | 0.478765 | 0.952331 | 0.025197 |
| IGLV4-69 | 0.89054 | 0.796581 | 0.995583 | 0.041572 |
| EFEMP2 | 1.389215 | 1.089223 | 1.77183 | 0.008085 |
| RAB3IL1 | 1.388183 | 1.008222 | 1.911337 | 0.044415 |
| HNRNPCP2 | 1.621687 | 1.022449 | 2.572124 | 0.039947 |
| G0S2 | 1.150428 | 1.010826 | 1.30931 | 0.033745 |
| PLAGL1 | 1.622695 | 1.214371 | 2.168316 | 0.001063 |
| ITM2A | 0.686466 | 0.526411 | 0.895186 | 0.005479 |
| MSRB3 | 1.40545 | 1.128033 | 1.751093 | 0.002415 |
| MMP1 | 1.15038 | 1.048704 | 1.261913 | 0.003005 |
| C18orf32 | 3.822337 | 1.355413 | 10.7792 | 0.011249 |
| BBS2 | 1.693579 | 1.049293 | 2.733469 | 0.031009 |
| SPINT1 | 1.770834 | 1.141431 | 2.747301 | 0.010762 |
| LSP1P4 | 1.470057 | 1.00301 | 2.154581 | 0.048226 |
| CNFN | 0.851666 | 0.742404 | 0.977008 | 0.021906 |
| IGKV3-15 | 0.885709 | 0.793195 | 0.989014 | 0.031066 |
